# Activated entomopathogenic nematode infective juveniles release lethal venom proteins

**DOI:** 10.1371/journal.ppat.1006302

**Published:** 2017-04-20

**Authors:** Dihong Lu, Marissa Macchietto, Dennis Chang, Mirayana M. Barros, James Baldwin, Ali Mortazavi, Adler R. Dillman

**Affiliations:** 1 Department of Nematology, University of California, Riverside, California, United States of America; 2 Department of Developmental and Cell Biology, Center for Complex Biological Systems, University of California, Irvine, California, United States of America; University of Pennsylvania, UNITED STATES

## Abstract

Entomopathogenic nematodes (EPNs) are unique parasites due to their symbiosis with entomopathogenic bacteria and their ability to kill insect hosts quickly after infection. It is widely believed that EPNs rely on their bacterial partners for killing hosts. Here we disproved this theory by demonstrating that the *in vitro* activated infective juveniles (IJs) of *Steinernema carpocapsae* (a well-studied EPN species) release venom proteins that are lethal to several insects including *Drosophila melanogaster*. We confirmed that the *in vitro* activation is a good approximation of the *in vivo* process by comparing the transcriptomes of individual *in vitro* and *in vivo* activated IJs. We further analyzed the transcriptomes of non-activated and activated IJs and revealed a dramatic shift in gene expression during IJ activation. We also analyzed the venom proteome using mass spectrometry. Among the 472 venom proteins, proteases and protease inhibitors are especially abundant, and toxin-related proteins such as Shk domain-containing proteins and fatty acid- and retinol-binding proteins are also detected, which are potential candidates for suppressing the host immune system. Many of the venom proteins have conserved orthologs in vertebrate-parasitic nematodes and are differentially expressed during IJ activation, suggesting conserved functions in nematode parasitism. In summary, our findings strongly support a new model that *S*. *carpocapsae* and likely other *Steinernema* EPNs have a more active role in contributing to the pathogenicity of the nematode-bacterium complex than simply relying on their symbiotic bacteria. Furthermore, we propose that EPNs are a good model system for investigating vertebrate- and human-parasitic nematodes, especially regarding the function of excretory/secretory products.

## Author summary

*Steinernema carpocapsae* belongs to a special group of insect-parasitic nematodes known as entomopathogenic nematodes (EPNs). These differ from other insect parasites in at least two ways; first they kill their hosts quickly (within 2–3 days), and second they associate with bacteria to facilitate their parasitic lifestyle. The infective stage of these parasites, the infective juvenile (IJ) stage, is the only free-living stage and these IJs are developmentally arrested and only reinitiate development once they are inside a suitable host. Little is known about the early stages of parasitism and how these parasites initiate the parasitic phase of their life cycle and reinitiate development. Here we characterized the changes that occur to the nematodes' physical morphology, gene expression, and the release of protein molecules that accompany the transition from developmentally arrested IJ to active, developing parasite. We showed that contrary to long-held assumptions, the nematodes are not merely transporting pathogenic bacteria but that the nematodes contribute to parasitism by releasing toxic proteins into the host. Many of the *S*. *carpocapsae* toxins are also found in species of human-parasitic nematodes, and *S*. *carpocapsae* may serve as a valuable model for understanding the specific function of these toxins.

## Introduction

Hundreds of millions of people are infected with parasitic nematodes worldwide [1, 2]. The immunomodulatory and pathogenic properties of parasitic nematodes are largely attributed to the excretory/secretory (ES) products they release during infection [3, 4]. ES products are complex mixtures and often include small molecules, proteins, and nucleic acids. The complexity of these products and technical limitations in obtaining sufficient quantities for separation studies have resulted in current efforts often being focused on the most abundant components [Reviewed in 4]. Some functional studies evaluated individual ES components and have produced provocative results in animal models as vaccine candidates and as potential therapeutics in autoimmune diseases [5, 6]. However, there are hundreds of identified ES products and few of them have been studied in any mechanistic detail. One major hindrance for mechanistic studies is the difficulty and cost in working with some vertebrate-parasitic nematodes and their hosts. Using model systems is a powerful way to discover conserved biology and to rapidly develop and test hypotheses [7]. Entomopathogenic nematodes (EPNs) are closely related to important species of human-parasitic nematodes [8] and could serve as model systems for studying parasitic nematode biology [9, 10].

EPNs are lethal parasites of insects. They associate with highly pathogenic bacteria and together EPNs and their mutualistic bacteria kill their hosts within a few days, distinguishing them from other insect parasites that develop longer associations with their hosts [11–13]. Because EPNs associate with pathogenic bacteria, the exact contribution of the nematode to this mutualism has remained uncertain. One widely accepted assumption is that the nematodes serve primarily as vectors for the pathogenic bacteria and that it is the bacteria that suppresses host immunity and ultimately kills the host [12–15]. While this is a good model for EPNs in the genus *Heterorhabditis* [16], there is evidence that EPNs in the genus *Steinernema* contribute to host immune suppression and to host killing. For example, axenic infective juveniles of *S*. *carpocapsae* are capable of killing hosts [16–18]. Even the cell-free growth media used to culture axenic *S*. *carpocapsae* has a toxic effect on potential insect hosts, suggesting that *S*. *carpocapsae* ES products may have pathogenic effects [19, 20]. Another study has shown that while one single *S*. *carpocapsae* infective juvenile (IJ) is sufficient to kill a pine weevil larva (*Hylobius abietis*), the LD_50_ of the bacterial symbiont, *Xenorhabdus nematophila*, is 3500 cells [21]. An individual IJ only contains 20–200 cells of the bacteria, suggesting that there is pathogenic synergy when both the nematode and the bacteria are present [18, 22]. These data support the notion that *S*. *carpocapsae* nematodes actively contribute to host immune suppression and host killing.

A number of studies have identified individual *S*. *carpocapsae* ES proteins and reveal likely functions in tissue degradation and immune suppression of the host [23–32]. However, no secretomic studies for EPNs have yet been performed, and the ~10 ES proteins that have been studied to date have each been identified and studied individually outside the context of infection. Here we reported the secretome of *S*. *carpocapsae* and tested its activity *in vivo*. We developed an improved *in vitro* model of inducing the excretion/secretion of protein products and validated this model by comparing the transcriptional profile of *in vitro* activation of IJs with *in vivo* infections. We showed that many of the ES products of *S*. *carpocapsae* are conserved or have high sequence similarity with those of vertebrate-parasitic nematodes and developed *S*. *carpocapsae* as a model of ES product function for *Strongyloides stercoralis* and other parasitic nematodes.

## Results

### IJs undergo quantifiable morphological changes when they transition from free-living to parasitizing a host

**Fig 1:**
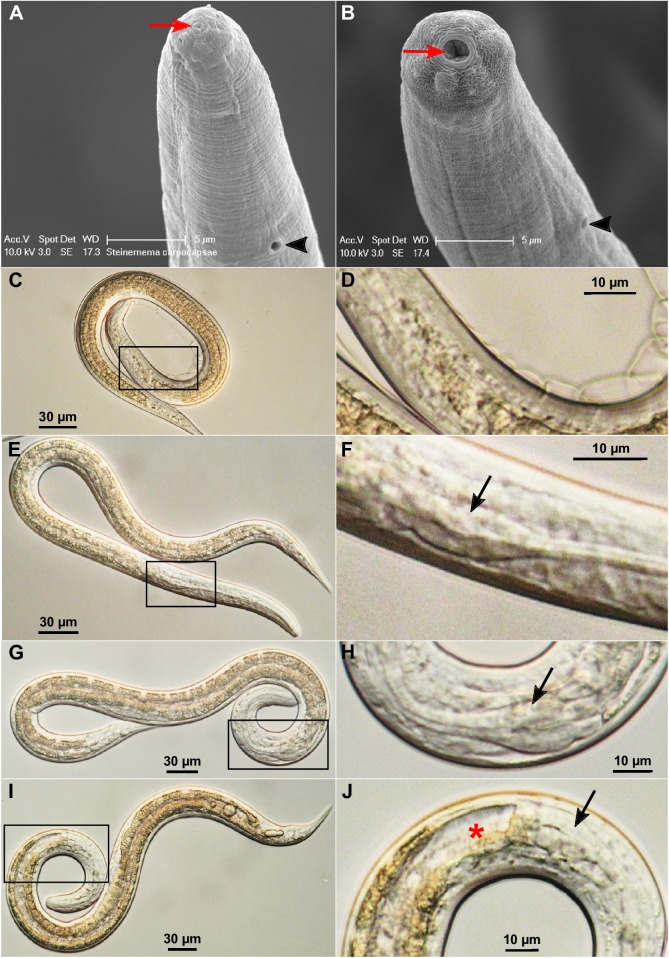
Morphological changes of activated *S*. *carpocapsae* nematodes. **(A)** The head region of a non-activated IJ by scanning electron microscopy (SEM). **(B)** The head region of an activated nematode by SEM. The mouth is marked by a red arrow and the excretory pore by a black arrowhead in (A) and (B). **(C)** A non-activated IJ by light microscopy (400x). **(D)** An enlarged view of the boxed region in (C). **(E)** A partially activated nematode (400x). **(F)** An enlarged view of the boxed region in (E) where the partially expanded terminal pharyngeal bulb (arrow) is located. **(G)** A fully activated nematode (400x). **(H)** An enlarged view of the boxed region in (G). **(I)** A fully activated nematode whose gut is wide open (400x). **(J)** An enlarged view of the boxed region in (I). The black arrowhead in (A) and (B) point to the secretory/excretory pore. The black arrows in (F), (H) and (J) point to the pharyngeal bulbs. The red star in (J) marks the open gut.

**Fig 2:**
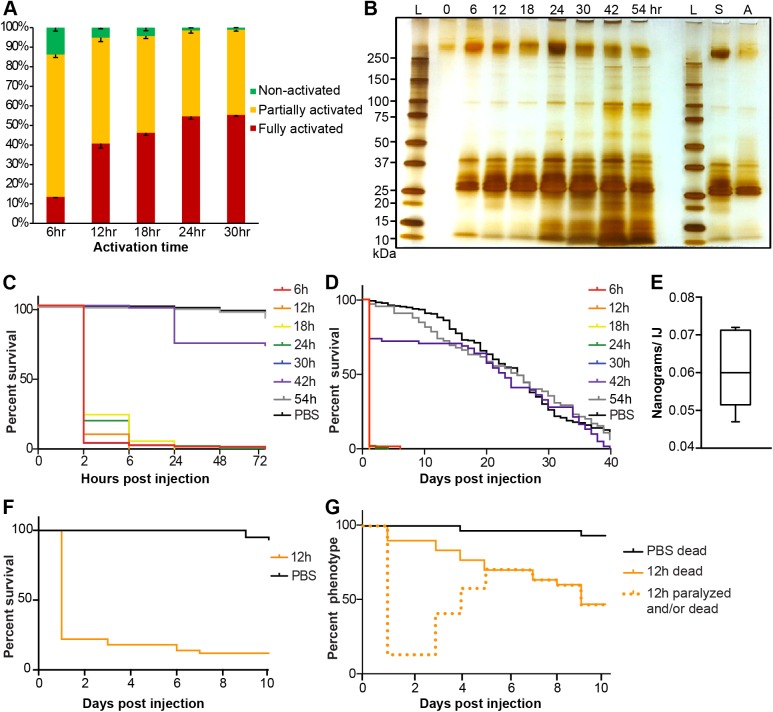
Activated *S*. *carpocapsae* ESPs contain lethal proteins. **(A)** *In vitro* activation rates of IJs over a time course. Error bars represent standard errors. **(B)** A silver-stained protein gel of ESPs from nematodes that have been activated for different lengths of time, and ESPs from axenic nematodes. The left-hand side of the gel shows the secreted proteins from symbiont-associated IJs activated for different amounts of time. The right-hand side of the gel shows the proteins secreted from symbiont-associated (S) and axenic (A) IJs that were exposed to waxworm homogenate for 12 h. Each lane contains 1% of the total ESP volume. The 0h sample was collected from non-activated IJs. L, protein ladder; S, symbiotic IJs; A, axenic IJs. **(C)** Survival curves within 72 hrs of *Drosophila* injected with 20 ng of ESPs from nematodes having been activated for different lengths of time. The 30 hr curve (blue) mostly overlaps with the 6 hr curve (red). **(D)** Survival curves over 40 days of *Drosophila* injected with 20 ng of ESPs from nematodes having been activated for different lengths of time. **(E)** The average amount of ESPs secreted by individual IJs in 3 hours. **(F)** Survival curves of 2^nd^ instar *Bombyx mori* larvae injected with 650 ng of ESPs from axenic nematodes that were activated for 12 h. **(G)** Phenotype curves of last instar *Galleria mellonella* larvae injected with 4 μg of ESPs from axenic nematodes that were activated for 12 h. The dotted line shows the number of larvae that were either killed or paralyzed after the injection. Paralyzed waxworms recovered over time.

Infective third-stage larvae (L3) or infective juveniles (IJs) of many parasitic nematodes are in a suspended stage of development [33, 34]. When IJs enter an appropriate host they undergo striking morphological changes and resume development [35, 36]. The resumption of feeding and development and the initiation of infection have been studied in detail in some parasitic nematodes, including *Ancylostoma caninum*, *A*. *ceylanicum*, *A*. *duodenale*, *Necator americanus*, and *Nippostrongylus brasiliensis* [35–41]. Entomopathogenic nematode infective juveniles are non-feeding with a sealed mouth and a collapsed pharynx [12, 42]. Similar to some animal-parasitic nematodes, when EPN IJs enter a host, as yet undefined signals induce recovery from the developmentally arrested IJ stage. This transition from free-living IJ to an active parasitic life style has been called many things; reactivation [39], resumption of feeding [36], resumption of development or initiation of development [35, 39], recovery [43], and activation [36, 41]. Because this transition from free-living IJ to an active parasitic life style requires dramatic morphological and physiological changes, we refer to the process as IJ activation rather than recovery, as the process is called in *C*. *elegans* dauers [44]. In order to study the contribution of the nematode to parasitism and host pathology, we wanted to be able to quantify IJ activation. Morphological characters can be used to differentiate between non-activated and activated IJs, so we leveraged these features to quantify the process of IJ activation [45]. The closed and opened mouth of non-activated IJs and activated nematodes, respectively, can be clearly observed by scanning electron microscopy (SEM) (Fig 1A and 1B). Additionally, we have found that the morphological change of the terminal pharyngeal bulb is a reliable indicator of IJ activation using traditional light microscopy. The pharyngeal bulb in non-activated IJs is generally invisible or at least much more obscure (Fig 1C and 1D; S1 Video); whereas it becomes easily observable when partially (Fig 1E and 1F) or fully (Fig 1G to 1J) expanded. Pharyngeal pumping is not discernible in partially expanded pharyngeal bulbs (S2 Video) but is prominent in fully expanded ones (S3 video). We used these characters to define three separate activation states: (1) nematodes without an observable pharyngeal bulb are non-activated IJs (Fig 1C); (2) those with a partially expanded pharyngeal bulb are partially activated (Fig 1E); and (3) those with a fully expanded pharyngeal bulb are fully activated IJs (Fig 1G and 1I). Although the fully activated nematodes may show different degrees of anterior gut opening or expansion (Fig 1G and 1I) and may even develop into L4 or young adult stages after activation for 18 hours or longer, they are not further subcategorized. The removal of the cuticle sheath (exsheathment) from L2 stage was not found to be a consistent indicator of activation. For example, we found that 31% of naïve IJs are already exsheathed (S1 Table) without activation and a small number of activated nematodes maintain the cuticle sheath.

### IJs activate in a time-dependent manner and activated IJs release a variety of proteins

Next we examined the activation of *S*. *carpocapsae* IJs over a time course (6 hr, 12 hr, 18 hr, 24 hr, and 30 hr) to determine the temporal activation rates (Fig 2A, S2 Table). When the IJs were incubated with waxworm homogenate for 6 hr, the total activation rate (partial and full activation) was already very high (86%). The total activation rate increased over time to almost 99% at the 30 hr time point. This demonstrates that IJs initiate the developmental process quickly once they are exposed to host tissue. Unlike the early plateau of the total activation rates, the percentage of IJs that fully activated increased gradually from 13.5% at 6 hr to ~55% at 24 and 30 hr time points. The observation that nematode activation is not synchronous *in vivo* coupled with the protein secretion data (Fig 2B) suggests that each IJ will be secreting a different arsenal of proteins, depending on the timing and its degree of activation. The nonsynchronous activation of *S*. *carpocapsae* IJs may be an example of phenotypic plasticity.

We then wanted to determine whether the process of IJ activation led to an output of excreted/secreted proteins (ESPs), which have been previously implicated in the parasitism of *S*. *carpocapsae* and other parasitic nematodes [e.g. 6, 29, 32, 46–48]. To do this we activated large batches of IJs in a time course, 6 hr, 12 hr, 18 hr, 24 hr, 30 hr, 42 hr, and 54 hr (~2 million IJs/sample; 3 replicates for each time point) and collected the ESPs. We showed that the non-activated IJs of *S*. *carpocapsae* secreted few if any proteins (Fig 2B). To determine whether the activated nematodes secrete different compositions of proteins over time, the same percentage (1%) of total ESPs from each time point was separated by a protein gel and visualized by silver staining. The qualitative profiles of abundant protein bands were similar for ESPs secreted at different time points, although the band intensities may vary due to different total ESP amounts and/or differential expression of certain proteins. We also found that IJs associated with *X*. *nematophila* and axenic IJs had similar profiles of high abundance secreted proteins (Fig 2B).

### The ESPs from activated IJs are highly toxic

Knowing that activated IJs release a diversity of ESPs into their hosts led us to wonder about the contribution of these proteins to the pathogenicity of the nematode/bacterial complex. We tested the toxicities of ESPs by injecting 5, 10, and 20 ng of concentrated proteins into adult *Drosophila* flies, which are a good model for studying EPN host-parasite interactions [49, 50]. We found that the ESPs from nematodes activated for 6–30 hr were consistently lethal to flies within 2–6 hr post-injection at a dose of 20 ng/fly (Fig 2C and 2D), but were not toxic at the dose of 5 ng/fly (S1 Fig). The toxicity of 10 ng/fly was less consistent between different protein samples (S1 Fig). Therefore, the dose of 20 ng/fly was used for all toxicity assays presented in Fig 2C and 2D. The 42 hr protein collection had much reduced toxicity (Fig 2C and 2D) and the 54 hr ESPs had no toxicity although the protein amounts and profiles were very similar to the 6–30 hr ESPs (Fig 2B), which indicates that the toxicity is mostly due to low-abundance proteins. Due to the highly toxic nature of these ESPs and their rapid deployment, we refer to them as venom proteins.

One lingering question was whether the activity we are seeing is due solely to nematode venom proteins and not toxins from the bacterial symbiont, *X*. *nematophila*. For all venom collection experiments we used a mixture of antibiotics (penicillin, streptomycin, and neomycin) to kill or suppress bacteria during IJ activation, washed activated nematodes thoroughly before venom collection, and filter-sterilized the collected proteins, there was still the possibility that the toxicity might result from a small amount of *X*. *nematophila* bacterial toxins. To address this, we obtained millions of axenic IJs by culturing them with a colonization defective mutant strain of *X*. *nematophila* [51] (S2 Fig). Several batches of 1000 axenic IJs were tested for their bacterial content. Unlike the symbiotic IJs, whose bacteria formed a lawn within 1 day, our axenic IJs did not show any bacterial growth even after 3 days, which confirmed that these IJs were indeed axenic (S2 Fig). We performed three replicates of activation using axenic IJs and collected the venom proteins. The axenic IJ venom had a similar protein profile and toxicity compared to symbiotic IJ venom (Fig 2B–2D, S1 Fig). This demonstrated that the activated nematodes indeed secrete lethal venom proteins. In addition to testing the toxicity of venom from axenic IJs in flies, we also injected them into silkworm larvae and waxworm larvae. We measured the weight of individual adult flies, 2^nd^ instar silkworm larvae, and last instar waxworm larvae to be approximately 0.8 mg, 20 mg, and 200 mg, respectively. We injected proportionately larger doses of venom in the silkworm and waxworm larvae, as they were considerably larger than adult fruit flies. We found that 650 ng of venom rapidly killed 2^nd^ instar silkworm larvae (Fig 2F). We found that 4 μg of venom appeared to rapidly kill waxworm larvae as well, as the larvae became unresponsive just hours after injection (Fig 2G). However, unlike what we observed with flies and silkworms, overtime, the waxworm larvae appeared to at least partially recover from the initial paralysis induced by the venom proteins (S4–S7 Videos), but they remained lethargic and less responsive than control animals (S4–S7 Videos). They also retained large dark spots near their heart, which we presume are the result of melanization (S3 Fig; S4–S7 Videos).

We then wanted to determine the ecological relevance of the amount of protein that was being secreted by activated IJs. Collected venom proteins were measurable by Bradford assay after ~500-fold concentration. Usually ~100 μg of venom proteins were obtained from ~2 million activated nematodes. We calculated that a single activated IJ secreted 0.061 ng of venom proteins within 3 hr (Fig 2E, S3 Table), and estimated that one IJ could secrete 0.49 ng proteins within 24 hr. Based on this estimation, we calculated that ~20 IJs are required to secrete 10 ng of venom proteins, the minimum lethal dose for *D*. *melanogaster*. This is an estimation based on the assumption that IJs continue to secrete protein once they are activated. Our data indicate that activated IJs do continue to secrete proteins for at least up to 54h after activation (Fig 2B) and that those products secreted between 6h and 30h of activation have a lethal effect on *D*. *melanogaster* adults (Fig 2C and 2D). The lethal dose will likely vary based on the host as we have shown in the case of waxworms and silkworms; it is possible that lower doses are effective against some natural hosts.

### Transcriptional analyses reveal similarity between *in vitro* and *in vivo* activation

We have shown that *in vitro* activated *S*. *carpocapsae* IJs release toxic venom proteins. The reason for doing *in vitro* activation rather than *in vivo* activation is that millions of IJs are required to generate enough venom proteins for downstream toxicity and proteomics analysis, a scale that cannot be achieved using *in vivo* infections. However, we did want to assess whether our *in vitro* activation method was a good proxy for what happens during an infection *in vivo*.

To test whether the *in vitro* IJ activation mimics the *in vivo* process, we compared the expression profiles of individual IJ-stage worms that were activated using these two methods. Specifically, we performed RNA-seq on individual non-activated IJs, 12h *in vitro* fully activated IJs, and IJs that were fully activated for 9 hr, 12 hr, and 15 hr *in vivo* using *Galleria mellonella* hosts. We chose to sequence the 12 hr *in vitro* activated IJs for three reasons: (1) they have a higher ratio of fully activated nematodes than IJs activated for 6 hr (Fig 2A), (2) the 12 hr fully activated nematodes are still activated IJs while longer activation times lead to more developed nematodes, and (3) their venom proteins were highly toxic (Fig 2C and 2D). We sequenced a time course of IJs activated *in vivo* to compare our *in vitro* activation and to characterize transcriptional dynamics during early infection.

**Fig 3:**
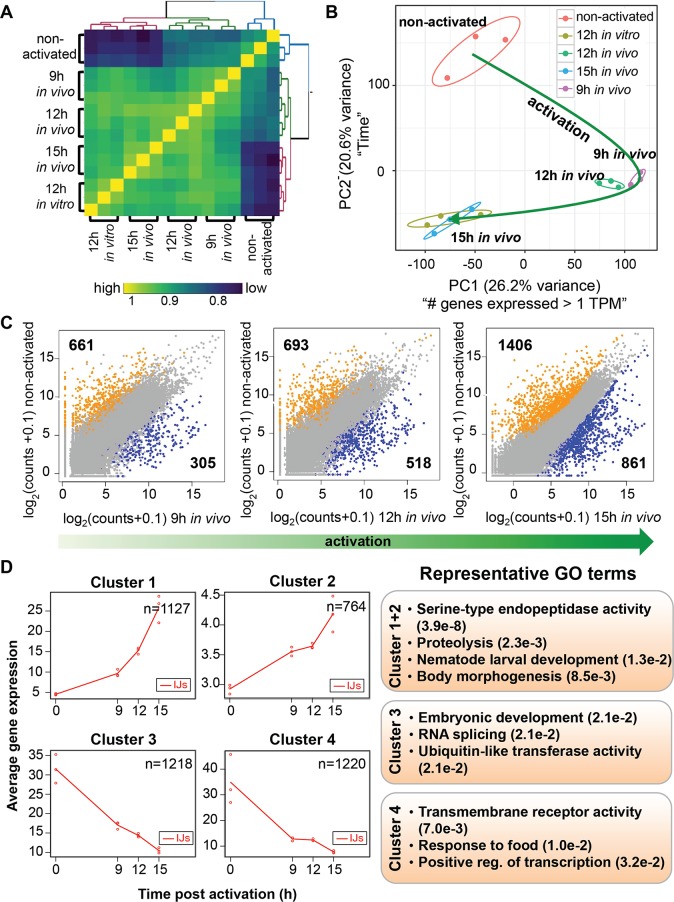
Gene expression dynamics during *in vivo* IJ activation. **(A)** Gene expression correlation matrix comparing the Spearman’s rank correlation coefficients between activated and non-activated IJs. Yellow colored cells indicate high correlations while blue cells indicate low correlations. Gene expression was transformed (log_2_(counts+1)) prior to calculating the correlation coefficients. Samples were K-means clustered by their Spearman’s rank correlation coefficients. **(B)** Activated and non-activated IJ transcriptomes plotted in the space of the first two principal components (which comprises 46% of the variation). A short description of what each principal component (PC) explains is included next to the PCs. The green line show the trajectory of IJ activation. **(C)** Scatterplots comparing the expression of all genes between non-activated IJs and 9 hr, 12 hr, 15 hr *in vivo* activated IJs. Genes labeled in orange and blue are differentially expressed (FDR < 0.05 and fold change > 2) between the stages compared, while grey genes are not. Expression for each gene was averaged across the biological replicates. **(D)** maSigPro plots showing the average expression of clusters of genes that are differentially expressed over the *in vivo* activation time course. The GO terms are representative of these expression clusters.

We generated a Spearman’s rank correlation matrix to determine the correlations of replicates and samples with each other (Fig 3A). In general, we found that IJs activated with both methods were more transcriptionally similar to each other than to the non-activated IJs, with 9 hr and 12 hr *in vivo* samples correlating better with each other (correlation ranges from 0.88 to 0.95) and 12 hr *in vitro* and 15 hr *in vivo* samples correlating better with each other (correlation ranges from 0.90 to 0.93). Principal component analysis of the IJs also supports the sample relationships we observed in the Spearman correlation matrix (Fig 3B), where we see 12 hr *in vitro* and 15 hr *in vivo* samples clustering with each other. Principle Component 1 appears to recapitulate the trends seen when the number of genes expressed > 1 TPM were plotted against the samples, where the 9 hr and 12 hr *in vivo* activated IJs have the fewest number of expressed genes whereas Principle Component 2 clearly corresponds to time (Fig 3B and S4 Fig).

Next, we performed differential gene expression (DE) analyses to determine genes that change expression during the early-stage of *in vivo* infection. We observed an increase in the numbers of DE genes over the *in vivo* time course, with more DE genes being downregulated than upregulated (1,406 genes versus 861 genes at 15 hr, respectively) (Fig 3C). To take the DE analysis a step further, we used maSigPro to find clusters of genes that are DE over the entire *in vivo* time course. We detected a total of 4,329 (15.2%) genes that change over the *in vivo* time course and generated four gene clusters with distinct dynamic profiles (Fig 3D); two clusters that increase over the time course (Cluster 1 and 2) and two that decrease over the time course (Cluster 3 and 4). We found slightly more genes decreasing (2,438 genes) during activation than increasing (1,891 genes), recapitulating the DE analysis results. We investigated the genes that are enriched in each cluster and found that genes involved in proteolysis and development increase during activation supporting the results of other studies [27, 52], whereas genes involved in transmembrane receptor activity (GPCRs), response to food, and positive regulation of transcription decrease during the time course (FDR < 0.05) (S5 Fig).

**Fig 4:**
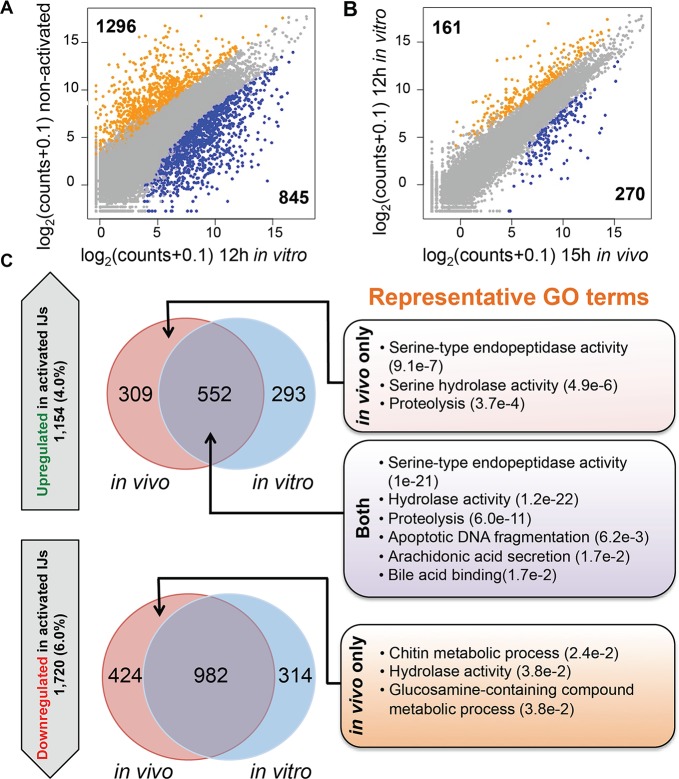
The gene expression profile of the 12 hour *in vitro* activated nematodes is most similar to that of 15 hour *in vivo* activated nematodes. **(A)** Scatterplot comparing the average gene expression counts of non-activated IJs and 12 hr *in vitro* activated IJs. Genes colored in orange and blue are differentially expressed (FDR < 0.05 and fold change > 2) and have higher expression in non-activated IJs and 12 hr *in vitro* activated IJs respectively. **(B)** Scatterplot comparing the average gene expression counts between 12 hr *in vitro* and 15 hr *in vivo* activated IJs. Genes colored in orange and blue are differentially expressed (FDR < 0.05 and fold change > 2) and have higher expression in 12 hr *in vitro* and 15 hr *in vivo* activated IJs respectively. **(C)** Venn diagrams showing genes that are upregulated and downregulated in the 12 hr *in vitro* and 15 hr *in vivo* activated IJs relative to non-activated IJs. The total number of upregulated and downregulated genes and their genome percentages are shown in the upward and downward block arrows, respectively. Representative GO terms and p-values for each category are listed on the right. Categories that do not have GO terms had no significant enrichment results.

To provide further evidence that *in vitro* and *in vivo* activated IJs are similar, we compared DE genes of the 12 hr *in vitro* activated IJs with the DE genes of the non-activated IJs and the IJs that had been activated *in vivo* for 9 hr, 12 hr, and 15 hr (Fig 4, S6 Fig). The number of DE genes between the 12 hr *in vitro* activated nematodes and non-activated IJs (1,296 down- and 845 upregulated genes; Fig 4A), and those between the 15 hr *in vivo* activated nematodes and non-activated IJs (1,406 down- and 861 upregulated genes) are comparable, and only a small number of genes differ between the 12 hr *in vitro* and the 15 hr *in vivo* activated nematodes (Fig 4B). Examination of genes that are upregulated through activation (15 hr *in vivo* only, 12 hr *in vitro* only, or both) relative to the non-activated IJs revealed genes involved in serine-type endopeptidase activity, proteolysis, and arachidonic acid secretion, which are activities associated with the IJ stage (Fig 4C, S7 Fig). We found no significant enrichment of GO terms for the set of genes that are downregulated in both activated IJs versus the non-activated IJs (982 genes). However, we did find that genes downregulated in only *in vivo* activated IJs are enriched in GO terms such as hydrolase activity and chitin metabolic process (S7 Fig).

### Nematode venom consists of a complex mixture of proteins

Since the venom released by activated nematodes is toxic, we wanted to determine the protein composition and to identify proteins that are potentially involved in parasite toxicity and immunomodulation of the host. Using mass spectrometry we identified 472 proteins released by the 12 hr *in vitro* activated nematodes with false discovery rates (FDRs) <1% (S4 Table). We were not only interested in the identities of these proteins, but also their corresponding gene expression levels during infection. We plotted the correlation between transcript levels (12 hr *in vitro*) and protein levels for the top 100 expressed proteins and found a positive correlation of 0.34 (Spearman’s rank correlation coefficient) (S8 Fig). However, these data confirm the fact that changes in RNA expression do not always equate to changes in protein translation.

**Fig 5:**
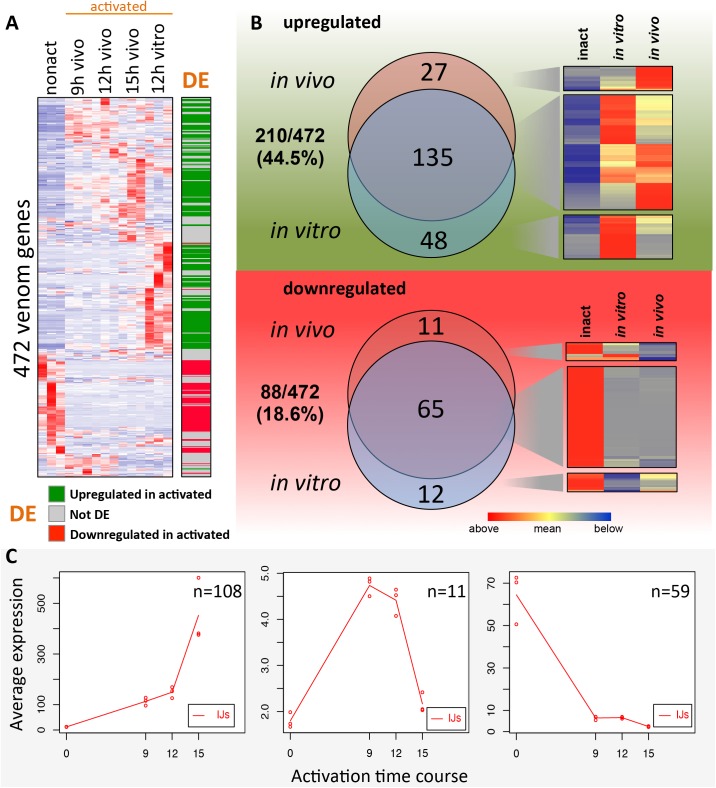
Expression of venom protein genes during IJ activation. **(A)** A heatmap showing the mean-centered gene expression counts of 472 venom protein genes across activated and non-activated IJs. Protein products of these genes were detected with mass spectrometry in the venom from 12 hr *in vitro* activated IJs. DE, differentially expressed. The green and red colored rows in the DE column represent the upregulated and downregulated genes in activated IJs, which are used in (B). **(B)** Venn diagrams showing the venom protein genes that are transcriptionally upregulated or downregulated in activated IJs relative to non-activated IJs, and their breakdown by activation method (12 hr *in vitro* and 15 hr *in vivo*). **(C)** maSigPro plots showing venom protein genes that are differentially expressed over the 15 hr *in vivo* activation time course.

We generated a heatmap of venom gene expression over the activation time course (Fig 5A). We found that 88/472 venom genes exhibited higher expression in the non-activated IJs in comparison to activated IJs (DE analysis: FDR < 0.05 and fold change > 2X) (Fig 5A and 5B). This indicates that these venom proteins may be synthesized before (preloaded) or quickly after IJ activation and immediately deployed upon host invasion.

The venom genes that are upregulated (Fig 5A, green in the DE column) and downregulated (Fig 5A, red in the DE column) in the 12 hr *in vitro* and 15 hr *in vivo* activated nematodes were compared to determine the similarities between the two samples (Fig 5B). Among the 210 upregulated venom genes, 135 (64%) are shared by the *in vitro* and *in vivo* samples (Fig 5B upper panel). Among the 88 downregulated genes, 65 (74%) are shared by the *in vitro* and *in vivo* samples (Fig 5B lower panel). The finding that the majority of differentially expressed venom genes are shared by the *in vitro* and *in vivo* activated nematodes strongly suggests that the *in vitro* activation method we have developed mimics the *in vivo* IJ activation process, especially venom secretion.

The expression dynamics of the 472 venom genes were further investigated using maSigPro (Fig 5C). During the time course 178 venom genes were DE throughout the *in vivo* activation time course and were grouped into three unique expression clusters. A set of 108 genes increased expression during activation, with a large spike between 12h and 15h (Fig 5C, left panel). The expression levels of 11 genes peak at 9–12 hr and then quickly decrease (Fig 5C, middle panel). Some of these genes include pore-forming proteins, proteases, lipases, and proteases that have annotations for cell death and may be involved in damaging host tissue. Another set of 59 venom genes decrease sharply in expression by 9 hr post activation.

We find it worth noting that 79 of the 178 (44%) DE venom genes are clustered in small groups (< 6 genes per group) with a mean of 2.54 genes per group (note: genes were clustered close together if they were within 10 genes of each other), suggesting that a substantial number of venom protein genes have a higher order organization in the genome and may be grouped into operons. This is consistent with the hypothesis that fast response genes such as those that would be turned on after recovery from the developmental arrest of dauers or IJs, may often be organized in operons for efficient use of limited amounts of RNA polymerase II [53].

**Fig 6:**
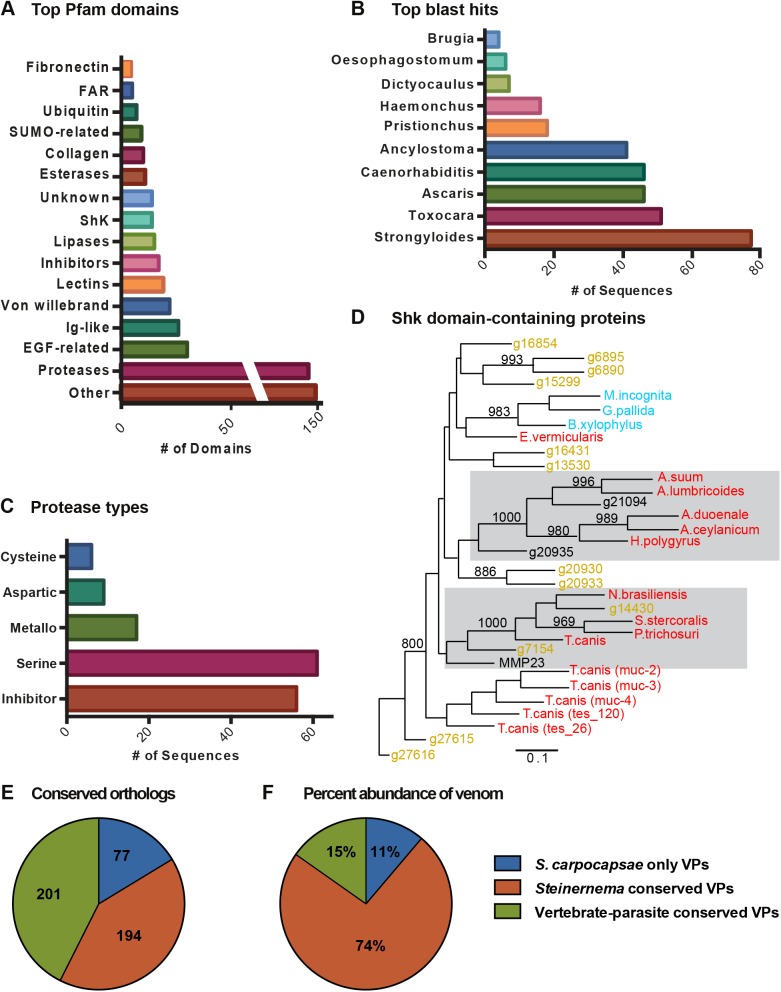
Comparative analysis of venom proteins from activated IJs. **(A)** Top BLAST hits of the 472 venom proteins in non-*Steinernema* organisms. **(B)** The top 16 Pfam domains in the venom proteins. **(C)** Merops breakdown of proteases and protease inhibitors found in the venom. **(D)** A neighbor-joining gene tree of the Shk domain-containing proteins found in the venom. Gray boxes highlight *S*. *carpocapsae* proteins with high similarity to Shk domain-containing proteins of vertebrate-parasitic nematodes. Bootstrap values of more than 80% (from 1000 replicates) are indicated at nodes. Bar, 10% divergence. **(E)** A pie chart showing the number of venom proteins that are conserved with at least one vertebrate-parasitic nematode, conserved with at least one other species of *Steinernema*, or are specific to *S*. *carpocapsae*. **(F)** A pie chart showing the percentage of molecules in the venom that belong to each of the categories above (conserved with at least one vertebrate-parasitic nematode, conserved with at least one other species of *Steinernema*, or specific to *S*. *carpocapsae*).

We used various bioinformatics tools to dissect the venom protein composition. We searched homologous proteins through BLAST to identify the highest sequence similarity to non-*Steinernema* genes. Fig 6A shows the 10 species with the highest number of matches to the venom proteins. Eight of these 10 species (*Brugia*, *Oesophagostomum*, *Dictyocaulus*, *Haemonchus*, *Ancylostoma*, *Ascaris*, *Toxocara*, and *Strongyloides*) are vertebrate-parasitic nematodes. The finding that these venom proteins are most similar to proteins found in vertebrate-parasitic nematodes suggests that the venom proteins of *S*. *carpocapsae* may be involved in parasitism rather than in common developmental pathways shared with free-living nematodes, though this remains to be tested. It also suggests that *S*. *carpocapsae* and related EPNs may be good model systems for studying vertebrate- or even human-parasitic nematodes and their secreted products.

We analyzed the venom protein Pfam domains to predict their functions (Fig 6B). Proteases and protease inhibitors are particularly abundant in the venom (Fig 6C). We also calculated how many members of each protein family encoded by the genome were secreted in the venom (Table 1). For example, 56% of the TILa domain-containing proteins in the *S*. *carpocapsae* genome are detected in the venom (Table 1). Large proportions of proteases (38% of serine carboxypeptidases, 36% of trypsins, 23% of eukaryotic aspartyl proteases, and 18% of zinc carboxypeptidases) and protease inhibitors (33% of trypsin inhibitor-like cysteine-rich domain and 19% of Kunitz/Bovine pancreatic trypsin inhibitor domain) contained in the genome are found in the venom. 17% of Shk domain-containing proteins that are predicted to be toxins [4, 46] are present in the venom. A gene tree was constructed for Shk domain-containing proteins from *S*. *carpocapsae* and other parasitic nematodes (Fig 6D). We found that several *S*. *carpocapsae* Shk domain-containing proteins had high similarity to those of vertebrate-parasitic nematodes, implying potentially conserved functions in parasitism (Fig 6D).

**Table 1:** A large proportion of some protein families are secreted venom proteins.

Pfam Domain	Pfam domain	# in Genome^a^	# in Venom^b^	% in Venom^c^
TILa domain	PF12714.2	16	9	56%
Serine carboxypeptidase	PF00450.17	24	9	38%
Trypsin	PF00089.21	114	41	36%
Ubiquitin-2 like Rad60 SUMO-like	PF11976.3	17	6	35%
Trypsin inhibitor-like cysteine-rich domain	PF01826.12	79	26	33%
Von Willebrand factor type A domain	PF00092.23	33	10	30%
Ubiquitin family	PF00240.18	29	7	24%
Eukaryotic aspartyl protease	PF00026.18	39	9	23%
Kunitz/Bovine pancreatic trypsin inhibitor domain	PF00014.18	43	8	19%
Zinc carboxypeptidase	PF00246.19	33	6	18%
Shk domain-like	PF01549.19	85	14	17%

^a^ The number of genes whose protein products contain the specified domain.

^b^ The number of venom proteins with the specified domain.

^c^ The percentage = 100% x (# in venom) / (# in genome). The percentage represents the proportion of each gene family that encodes venom proteins.

Next we performed an orthology analysis to determine the conservation of venom proteins among nematodes. The orthology analysis included the 472 venom proteins and the proteomes of 13 other nematodes including 8 species of vertebrate-parasitic nematode, *Heterorhabditis bacteriophora*, and 4 other sequenced species of *Steinernema*. The venom proteins clustered into 321 clusters. We found that 43% of *S*. *carpocapsae* venom proteins share orthology with vertebrate-parasitic nematodes, but that the majority (57%) are specific to *Steinernema* (Fig 6E; S5 Table). In this orthology analysis we found that 168 *S*. *stercoralis* genes clustered with 148 of the 472 *S*. *carpocapsae* venom proteins into 116 total clusters. We assessed the gene expression profiles of these 168 *S*. *stercoralis* genes using previously published data [54], to determine whether these genes are expressed during parasitic stages. We found that the majority of these 168 *S*. *stercoralis* genes show expression in the infective juvenile (infectious 3^rd^ larval stage) or activated infective juvenile stages (tissue migrating 3^rd^ larval stage) (S9 Fig). We found that many of these genes are highly expressed in the infective stages with more than one third of them being expressed at levels over 1000 transcripts per million (TPM) and only 12 of these 168 *S*. *stercoralis* genes being expressed at levels lower than 10 TPM (S9 Fig).

Using the exponentially modified protein abundance index (emPAI) to estimate the absolute protein amount in our mass spectrometry data, we calculated the percentage of proteins in the venom that came from proteins conserved with vertebrate-parasitic species, those that were conserved with other species of *Steinernema*, and those that were specific to *S*. *carpocapsae*. We found that ~85% of the proteins in the venom were specific to *Steinernema* species (Fig 6F), indicating that although many of the venom proteins have orthologs in other parasites, *Steinernema*-specific proteins are the most abundantly secreted venom proteins.

## Discussion

To successfully establish parasitism, nematodes need to first find a proper host, enter the host, and then launch parasitic programs for survival. Host-seeking is a behavioral response of free-living IJs to host cues and is reversible if the IJs either fail to locate and enter a host or if subsequent cues lead to the decision not to enter the host [55]. The parasitic activation of IJs is irreversible once they have entered a host, and thus in the case of EPNs, activated nematodes are fully committed to killing the host. Understanding the critical transition from free-living IJ to active parasitic stages may reveal early mechanisms of host-parasite interactions and elucidate the biology underlying pathogenicity.

### *Steinernema* EPNs are more than pathogen vectors

EPNs are a special group of insect parasitic nematodes, which associate with symbiotic bacteria and kill insect hosts within a few days [11, 12]. The symbiotic bacteria have been extensively investigated and are highly toxic to insects, leading to a widely accepted assertion that the nematodes serve as vectors for the symbiotic bacteria and that the bacteria kill the hosts to provide food for the nematodes [13]. However, a few studies have demonstrated that axenic IJs of *S*. *carpocapsae*, but not of *H*. *bacteriophora* were able to kill insects that can support nematode growth and reproduction [16–18], and that even the growth medium of axenic *S*. *carpocapsae* was also lethal to hosts [19, 20], suggesting that the nematodes also contribute significantly to the pathogenicity of the nematode-bacterial complex rather than simply being a vector for the pathogenic bacteria. Simões *et al*. have identified 10 individual effectors from *Steinernema* EPNs [23–32]. These proteins have been shown to have potential functions in immune suppression and tissue damage. Our study has identified hundreds of putative venom proteins released by activated *S*. *carpocapsae* IJs using mass spectrometry. We have confirmed the toxicity of the complex mixture of released proteins in several insect hosts and show that these secreted proteins have high toxicity even when harvested from axenic nematodes. Injections of the venom proteins into last instar waxworms revealed a strong paralytic activity, from which many of the insects partially recovered. Our findings strongly suggest a new working model, at least for *Steinernema* EPNs, where both the nematodes and the symbiotic bacteria contribute to toxicity in hosts via secreted products.

The secretome and transcriptome data together suggest that a portion of the venom genes are highly transcribed in the host-seeking IJ stage and the venom proteins are possibly preloaded, ready to be released into the host immediately after activation. Further, our toxicity tests showed that 10 ng of venom proteins can kill *Drosophila* adults (S1 Fig), which is approximately the amount secreted by 20 nematodes over a 24 hour period. It may take more venom or a longer amount of time to kill larger hosts or hosts with stronger immune systems; or, the nematode venom alone may not kill the hosts in the natural environment but instead have synergistic effects with bacterial growth and metabolites, as previous studies suggested [21]. The fact that both adult flies and larval silkworms rapidly died but that most waxworm larvae were paralyzed rather than immediately killed reveals that the venom has different effects in different hosts or different insects have variable sensitivities to the venom. Further studies are required to reveal the details of the host-parasite antagonism and the relative roles of nematode and bacteria.

Our mass spectrometry results showed that the nematode venom is a complex mixture of proteins. Although we focused on proteins, we are aware of the possibility that other molecules may also be present in the venom, such as nucleic acids or small metabolites [56, 57], which can be analyzed by different mass spectrometry techniques. Additional investigation and identification of specific active components of EPN venom will be essential for understanding how the nematodes interact with their hosts. It is not only important in the case of EPNs, but may also be informative for vertebrate- and human-parasitic nematodes. Previous studies have suggested that helminth suppression of inflammatory immune responses in human intestine may be harnessed to treat inflammatory bowel disease [6, 58]. To avoid the use of live nematodes in therapy, we need to identify the active molecules that are released by the nematodes to modulate the host immune system. These include proteins and possibly small molecules such as ascarosides [59], which we have not explored here. We found that many of the *S*. *carpocapsae* venom proteins have orthologs in vertebrate-parasitic nematodes. For example, we found 168 orthologs to the *S*. *carpocapsae* venom proteins in *S*. *stercoralis*. Further, we found that all of these 168 *S*. *stercoralis* orthologs are expressed, many of them highly expressed in infective stages, leading us to speculate that these are putative venom proteins in *S*. *stercoralis*. However, unlike vertebrate-parasitic nematodes, EPNs rapidly kill their hosts. We found that the bulk of the total secreted venom proteins we analyzed consisted of *Steinernema*-specific proteins. We have not yet identified which protein(s) are actively causing death or paralysis, but our data suggest that it is the low abundance proteins that are responsible for these effects. Using EPN venom as a model for vertebrate-parasitic nematode ES products may facilitate the generation and testing of hypotheses in a way that is not currently possible in other systems.

### The IJ activation process can be studied *in vitro*

We found that the *in vitro* activation mimicked the *in vivo* process based on gene expression profiles. We hypothesized that the gene expression is temporally dynamic. Therefore, we did a time course transcriptome analysis of *in vivo* activation (9 hr, 12 hr, and 15 hr) and found that the 15 hr *in vivo* activated nematode transcriptome correlated best with the transcriptome of 12 hr *in vitro* activated nematodes. The extra 3 hr difference for *in vivo* activation may result from host seeking and penetration, which is not required for *in vitro* activation. The similarity between the *in vivo* and *in vitro* activation is encouraging and suggests that the *in vitro* conditions we have developed accurately mimic *in vivo* infection, at least in the early stages.

There were some differences in gene expression between the *in vivo* and *in vitro* activated nematodes. These could be due to the presence or absence of host immune response, differences in food quality and quantity, oxygen levels, or nematode population densities, etc. Although we tried to mimic the *in vivo* resources and texture by using insect homogenate absorbed by sponge pieces, some differences were inevitable. For example, (1) the insect homogenate was free of solid tissue; (2) the insect homogenate was heated to prevent melanization of hemolymph; and (3) the sponge, although providing aeration and tactile cues, was different from living insect cuticle in many aspects. Despite the technical differences, our results suggested that the essential genes and pathways for the IJ activation and pathogenicity are turned on or off in similar ways.

Variations in the transcriptomes of individual nematodes (biological replicates) may be due to technical and/or biological factors. Cutting and digesting the nematodes may cause loss of some cells. Genetic/epigenetic background and even the sex of individual IJs may differ. Although we confirmed the full activation status of the selected nematodes, they may not have been synchronized. It is common to incur variations using a small sample size. One potential way to reduce variation is to sequence more individual nematodes of the same activation condition, which could result in fewer distinct clusters of individual transcriptomes (e.g., due to sexes) or be averaged across the samples. Another way to reduce variation is to pool the selected nematodes from each condition, which, in addition to reducing variation may also obscure the transcriptional resolution we obtained by sequencing individual nematodes.

### EPNs are a good model system for studying vertebrate-parasitic nematodes

EPNs are a good model system for studying nematode-bacterium symbiosis, nematode-insect host interactions, and bacterium-insect host interactions. Discoveries about EPN biology are not only important for improving their efficacy as biological control agents, but are also important for understanding other parasitic nematodes, like those that cause diseases in mammals.

Much like other model systems that are widely used for studying numerous conserved biological processes related to human health, EPNs are increasingly considered good model organisms for studying vertebrate-parasitic nematodes. Some reasons that support their use as a model system include:

1. Vertebrate-parasitic nematodes and EPNs are closely related [8]. Thus, there is likely to be a high level of conservation between these groups. EPNs, like many vertebrate-parasitic nematodes are able to modulate host immunity [29, 30]. Therefore, EPNs with their broad range of infectivity strategies and likely differences in venom compositions may serve as a group of model organisms for understanding the role and function of certain secreted molecules in parasitism.
2. EPNs are technically easier and safer to culture and manipulate than vertebrate-parasitic nematodes. EPNs infect insects but not humans and therefore are less demanding in personal protection, research facilities, and host animals. They are easy and inexpensive to culture in large quantities in insects or *in vitro*. It is possible to culture them with or without their symbionts and the nematodes can develop through all life stages *in vitro*. We obtained millions of axenic *S*. *carpocapsae* IJs by culturing them on a colonization defective mutant of *X*. *nematophila* and were able to harvest enough venom proteins for toxicity analysis. This large-scale culture is extremely difficult, if not impossible in vertebrate-parasitic nematodes, most of which are not easy to culture *in vitro* and have not been shown to develop through all life stages *in vitro*. There are plenty of examples of proteomic studies of ES products from vertebrate-parasitic nematodes; however it remains uncertain whether the *in vitro* techniques used to generate the material actually mimic parasitism conditions *in vivo* [60–62]. In addition, it is relatively easy to collect EPNs of different developmental stages *in vivo* or *in vitro* for time-resolved analysis of transcriptome or proteome dynamics, such as our time course of *in vivo* IJ activation for transcriptome analysis and time course of *in vitro* IJ activation for testing venom toxicity. Such a fine temporal resolution is much more difficult to achieve in vertebrate-parasitic nematode systems.

## Conclusions

Our study of *S*. *carpocapsae* venom proteins and transcriptome dynamics during IJ activation leads to a new paradigm that EPNs also significantly contribute to toxicity in the host in addition to serving as vectors for entomopathogenic bacteria. We also found that EPN secretions contain many proteins that are conserved in other parasitic nematodes, providing new evidence to support the notion that EPNs and vertebrate-parasitic nematodes may use similar molecules to facilitate their parasitism. Thus, we propose that EPNs can be used as a model system for studying conserved aspects of vertebrate-parasitic nematodes, especially the bioactive components they release to manipulate the hosts.

## Materials and methods

### Insects

*Galleria mellonella* (waxworms) were purchased from CritterGrub (www.crittergrub.com). Healthy waxworms were sorted to remove wood chips, rinsed with tap water, surface dried on paper towels, and used immediately for infection by IJs or frozen at -80°C for making waxworm homogenate later.

*Bombyx mori* eggs were purchased from Mulberry Farms (www.mulberryfarms.com). Hatching larvae were fed silkworm chow, purchased from Mulberry Farms, *ad libitum*.

Oregon-R *Drosophila* flies were kept in standard fly bottles as previously described [63]. Briefly, flies were reared in bottles containing dextrose medium (129.4 g/L dextrose, 7.4 g/L agar, 61.2 g/L corn meal, 32.4 g/L yeast, and 2.7 g/L tegosept; polypropylene round bottom 8oz bottles plugged with bonded dense weave cellulose acetate plugs, Genesee Scientific Cat #49–100) and were housed at 25°C with 60% relative humidity and a 12 hr light and 12 hr dark cycle.

### Symbiotic and axenic *S*. *carpocapsae* IJs

Symbiotic IJs were produced by *in vivo* culture in waxworms. Twenty clean waxworms were put in a 10 cm Petri dish with a piece of filter paper on the bottom. Symbiotic IJs (~50 IJs/host) of *S*. *carpocapsae* (strain All) were suspended in 1 ml of tap water and distributed onto the filter paper. The infection can be scaled up to many plates to obtain more IJs. The infection plates were incubated at 25°C with 60% humidity in the dark for 2 weeks. Then the waxworm cadavers were transferred to White traps [64] in which a piece of 5.5 cm filter paper was raised by the lid of a 3.5 cm Petri dish in a 10 cm Petri dish with a thin layer of tap water (1–2 mm deep). Within 1–2 weeks the IJs gradually migrated into water. The IJs were collected and washed with tap water at least 3 times before being stored in tissue culture flasks with vented caps (VWR, 10062–860). Washing a large number of IJs was performed in a glass vacuum filter holder (Fisher Scientific, 09-753-1C) with two layers of 11 μm pore-sized nylon net filters (Millipore, NY1104700). Small populations of IJs were washed in 15 ml tubes by centrifugation at 700 rcf for 1 min at room temperature in a swing-bucket rotor. The supernatant was removed after each wash. The density of IJs was controlled to be lower than 15 IJs/μl to prevent oxygen depletion. The IJs were stored at 15°C.

Axenic IJs were produced by *in vitro* culture on the colonization defective mutant bacterial strain HGB315 of *X*. *nematophila* [51]. The mutant bacteria were streaked from a -80°C glycerol stock onto a NBTA plate (8.0 g/L nutrient agar; 25 mg/L bromothymol blue; 40 mg/L 2,3,5-triphenyltetrazolium chloride) supplemented with 0.1% (w/v) Sodium Pyruvate and a MacConkey plate (Difco MacConkey Agar, #212123) supplemented with 0.1% (w/v) Sodium Pyruvate. All colonies turned dark blue on the NBTA plate and reddish/brown on the McConkey plate (S2 Fig), confirming that they were the phase I bacteria. Mutant *X*. *nematophila* was inoculated in 5 ml of Luria-Bertani (LB) broth with 0.1% (w/v) Sodium Pyruvate and cultured overnight at 25–28°C with shaking at 220 rpm. 100–200 μl of overnight liquid culture was spread on lipid agar plates (8 g/L of nutrient broth, 5 g/L of yeast extract, 2 g/L of MgCl2, 7 ml/L of corn syrup, 4 ml/L of corn oil, and 15 g/L of Bacto Agar) supplemented with 0.1% (w/v) Sodium Pyruvate and incubated at 25–28°C for 24h or until a bacteria lawn formed. 1.5x10^4^ symbiotic *S*. *carpocapsae* IJs were seeded onto the mutant bacteria lawn. After 3 days, the nematodes developed to mature adults including gravid females, which were washed 3 times in a 15 ml tube with autoclaved 0.8% NaCl solution. The adult nematodes were treated twice by 15 ml bleach solution (0.5N NaOH and 0.5–0.6% NaClO) for 10 min each time. All the tissues were digested except the eggs/embryos (S2 Fig), which were washed 3 times by 0.8% NaCl solution and added to the mutant bacteria lawn on lipid agar plates. Nematodes hatched and developed to adults (S2 Fig), which were split to multiple mutant bacteria plates for obtaining more IJs.

Infective juveniles were washed off the mutant bacteria plates and washed 10 times with 0.8% NaCl solution. We surface sterilized 1000 IJs using 1 ml of 4 mM Hyamine 1622 solution (Sigma, 51126) for 30 min and washed 3 times with 0.8% NaCl solution. The IJs were homogenized in 50 μl of 0.8% NaCl solution by a tissue grinder (Fisher Scientific, 12-141-363) and plated on the LB plates supplemented with 0.1% (w/v) Sodium Pyruvate. The plates were incubated at 28°C in dark and checked for bacteria growth every day for 3 days. Four replicates were performed for the IJs from mutant bacteria plates and two replicates of 1000 symbiotic IJs were used as the control (S2 Fig).

### Waxworm homogenate preparation

Frozen waxworms (25 g for making 100 ml 25% homogenate) were ground in liquid nitrogen with a mortar and pestle into a fine powder, which was then transferred to a clean beaker. Phosphate-buffered saline (PBS: 137 mM NaCl, 2.7 mM KCl, 10 mM Na_2_HPO_4_, 1.8 mM KH_2_PO_4_, pH 7.4) was added to suspend the powder to reach the desired volume. The beaker was loosely covered with plastic wrap and heated in a microwave oven until the suspension started boiling. The suspension was stirred and microwaved to boil again. The boiling step was repeated 3–4 times in total. The homogenate was cooled to room temperature in a water bath. The contents were transferred to 50 ml tubes and centrifuged at 3000 rcf for 5–10 min at 25°C. The supernatant, including with the top lipid layer, was transferred to new tubes and mixed vigorously before being aliquoted and frozen at -20°C for future use. The homogenate was supplemented with 1x antibiotics immediately prior to use (penicillin, streptomycin, and neomycin solution; Sigma, P4083-100ML).

### Activation of IJs and venom collection

About 2 million IJs were washed at least 3 times with washing solution (autoclaved 0.8% NaCl solution containing 0.01% Triton X-100 which prevents IJs from sticking to plastic and glass surfaces) in a glass vacuum filter holder (Fisher Scientific, Cat# 09-753-1C) with two layers of 11 μm nylon net filters (Millipore, NY1104700). The IJ suspension was then transferred to a 15 ml tube and centrifuged at 700 rcf for 0.5–1 min at room temperature. The supernatant was removed leaving about 5 ml of suspended IJs. The IJs were transferred to a 1 L flask containing 8.5 g of autoclaved sponge pieces soaked with 100 ml of 25% waxworm homogenate and 1x antibiotics (Sigma, P4083-100ML). The sponge and IJs were incubated at 25°C in the dark for a certain amount of time (specified in the experiments) for IJ activation.

To recover the nematodes after incubation, the sponge pieces were soaked in a 1 L beaker containing about 500 ml of washing solution for 5–10 min with occasional stirring (squeezing the sponge was avoided since doing so could damage nematodes). The nematode suspension was transferred to another beaker and 500 ml of washing solution was added to the sponge. The soaking step was repeated 5–6 times to recover most of the nematodes. The nematodes were collected in the glass vacuum filter mentioned above. The solution was removed, leaving only a thin layer of solution covering the nematodes. Then 100 ml of washing solution was immediately added and the nematodes were resuspended by pipetting. This washing step was repeated 10 times to remove contamination. The clean nematodes were resuspended in 105 ml of PBS solution and transferred to a sterile 1 L glass flask. The flask was incubated in a shaker set to 25°C and 200 rpm for 3 hr for the nematodes to continue to release venom. Then the nematodes were pelleted in 15 ml tubes by centrifugation at 700 rcf for 0.5–1 min at room temperature. The nematodes were saved for quantification of activation rates and microscopy. The supernatant was filtered through a low protein binding 0.22 μm syringe filter (Fisher Scientific, Cat# 9719001) to remove residual nematodes and bacteria and collected in two 50 ml tubes (approximately 100 ml). The filtered supernatant containing the nematode venom was stored at -20°C or immediately concentrated in an Amicon Ultra 15 ml centrifugal 3kDa filter (Millipore, UFC900308). The centrifugal filter was filled with 15 ml of venom and centrifuged in a swing-bucket rotor at 3000 rcf at 4°C for 50–60 min to concentrate to less than 1 ml. The flow-through solution was discarded and another 15 ml of venom was added to the centrifugal filter. The centrifugation was repeated until the 100 ml of venom was concentrated to the dead volume of the filter (~200 μl). The concentrated venom proteins were transferred to a 1.5 ml low retention tube (Fisher Scientific, Cat# 21402903). Protein concentration was measured using a Bradford assay using the Bio-Rad protein assay dye reagent (Bio-Rad, 500–0006). The remaining concentrated venom was stored at -80°C. Three biological replicates of activation and venom collection were performed.

### Quantification of activation rates

After activation and venom collection, the nematodes were diluted to a density of 10–20 nematodes/μl for quantification of activation rates. Approximately 10 μl of nematode suspension was placed on a glass slide and covered by a cover slip for observation with a compound light microscope. 100x magnification was enough for identifying the partially activated and fully activated nematodes with partially and fully expanded pharyngeal bulbs, respectively (Fig 1E–1J). When the phenotype was difficult to score, higher magnification (200x or 400x) was used. Nematodes without obviously visible pharyngeal bulbs were considered as non-activated (Fig 1C and 1D). The quantification was repeated at least 3 times for each sample and 3 biological replicates were performed. Photos and videos of nematodes were taken using a Canon EOS T5i camera attached to a Leica DM2500 microscope.

### Scanning electron microscopy (SEM)

Non-activated IJs were treated by 0.5–0.6% sodium hypochlorite (10-fold dilution of household bleach containing 6% sodium hypochlorite) for 1 min to dissolve L2 cuticle sheath and immediately washed 3 times by 0.8% NaCl solution. Nematodes that were fully activated *in vitro* for 18h were washed 3 times by 0.8% NaCl solution. The washed non-activated IJs and activated nematodes were processed and observed by SEM as described previously [65].

### Protein electrophoresis

Proteins were denatured by SDS sample buffer (final 1x buffer: 50 mM TrisHCl pH6.8, 2% SDS, 2.5% Ficoll, 0.01% bromophenol blue, and 0.1 M DTT) and heating for 10 min in boiling water before being loaded to a 4–15% Mini-PROTEAN TGX Precast Gel (Biorad, # 4561086). Protein molecular weights were marked by the Precision Plus Protein Dual Color Standards (Biorad, #1610374). The electrophoresis was run at 100 Volts for 60–90 min and the gel was stained by the Pierce Silver Staining kit (Pierce, # 24600) following the manufacturers instructions.

### Venom toxicity assays

To test toxicity in flies, 5–7 day old male flies were sorted into new fly vials before being used for injection as previously described [63]. Flies were anesthetized with CO_2_. The concentrated venom proteins were diluted to 0.4 μg/μl (= 20 ng/50 nl), 0.2 μg/μl (= 10 ng/50 nl) and 0.1 μg/μl (= 5 ng/50 nl), 50 nl of which was injected into the anterior abdomen of each fly by a high-speed pneumatic microINJECTOR (Tritech Research, MINJ-FLY) and a pulled glass needle. Three biological replicates of venom were tested for toxicity. Three technical replicates of >20 flies each were performed for every biological replicate (i.e., >60 flies per biological replicate). Flies injected with PBS solution were used as the negative control. Injected flies were maintained in fly vials and their survival was recorded at 2 hr and 6 hr post-injection, and every 24 hr thereafter until all flies died. To test toxicity in silkworms, 2^nd^ instar larvae were anesthetized using CO_2_ and were injected with 1 μl of 0.65 μg/μl venom in one of the prolegs in the penultimate pair using a microINJECTOR (Tritech Research, MINJ-FLY). Silkworms were injected with 650 ng of venom from axenic *S*. *carpocapsae* IJs that had been activated for 12 h in waxworm homogenate. We injected 50 larvae with venom and 50 larvae with PBS buffer. Survival was recorded for 10 days. Injected larvae were fed silkworm chow *ad libitum*. To test toxicity of the venom in waxworms, last instar waxworm larvae were injected into one leg of the last pair of prolegs as previously described [66]. Each larva was injected with either 10 μl of PBS buffer or 10 μl of venom that was concentrated to 0.4 μg/μl for a total of 4 μg per larva. Waxworm larvae were injected using a 10 μl gastight syringe (Hamilton). We injected 30 larvae with venom and 30 larvae as a control. Injected larvae were monitored for 10 days but not fed. Survival curves were plotted with the GraphPad Prism software (GraphPad Software, Inc. La Jolla, CA).

### Protein digestion by trypsin/Lys-C

Venom proteins were digested by Trypsin/Lys-C Mix, Mass Spec Grade (Promega, V5072) following the product manual with some modifications. Then 30 μg of proteins were denatured in 6–8M Urea and 50mM Tris-HCl (pH 8). The disulfide bonds were reduced by 5 mM dithiothreitol (DTT) for 30 minutes at 37°C followed by alkylation by 15 mM iodoacetamide for 30 min at room temperature in the dark. Excess iodoacetamide was quenched by adding DTT to 10 mM (with the previously added 5 mM making the total 15 mM). Trypsin/Lys-C Mix was added to the protein sample at a 25:1 protein:protease ratio (w/w) and incubated for 3–4 hr at 37°C.

The reaction was diluted with 50 mM Tris-HCl (pH 8) to reduce the urea concentration to 1 M or below and incubated overnight at 37°C. Protein digestion was terminated by adding trifluoroacetic acid (TFA) to a final concentration of 0.5–1%, and particulate material was removed by centrifuging at 14,000–16,000 rcf for 10 minutes. The supernatant was transferred to a C18 spin column (Pierce, 89873) for peptide clean-up following the product manual. The resin in the column was rinsed twice by 200 μl of activation solution (50% acetonitrile) and centrifuged at 1500 rcf for 1 min. Then the resin was rinsed by 200 μl of equilibration/wash solution (0.5% TFA in 5% acetonitrile) and centrifuged at 1500 rcf for 1 min. The peptide sample was mixed with 1/3 volume of 2% TFA in 20% acetonitrile and loaded to the column and centrifuged at 1500 rcf for 1 min. The flow-through was reloaded to the resin and centrifuged again. The resin was then washed twice by 200 μl of equilibration/wash solution and centrifuged at 1500 rcf for 1 min. The column was placed in a new 1.5 ml tube and 20 μl of elution buffer (70% acetonitrile) was added to the resin bed that was centrifuged at 1500 rcf for 1 min. The elution was repeated one more time to collect a total of 40 μl of peptide solution that was dried in a vacuum evaporator.

### Mass spectrometry

A MudPIT approach was employed to analyze the trypsin-treated sample. A two-dimension nanoAcquity UPLC (Waters, Milford, MA) and an Orbitrap Fusion MS (Thermo Scientific, San Jose, CA) were configured together to perform online 2D-nanoLC/MS/MS analysis. 2D-nanoLC was operated with a 2D-dilution method that is configured with nanoAcquity UPLC. Two mobile phases for the first dimension LC fractionation were 20 mM ammonium formate (pH 10) and acetonitrile, respectively. Online fractionation was achieved by 5 min elution off a NanoEase trap column (Waters, 186003682) using stepwise-increased concentration of acetonitrile. A total of 5 fractions were generated with 13%, 18%, 21.5%, 27%, and 50% acetonitrile, respectively. A final flushing step used 80% acetonitrile to clean up the trap column. Every fraction was then analyzed online using a second dimension LC gradient. The second dimension nano-UPLC method was described previously [67].

Orbitrap Fusion MS method was based on a data-dependent acquisition (DDA) survey. The acquisition time was set from 1–70 min. Nano ESI source was used with spray voltage at 2600V, sweep gas at 0, and ion transfer tube temperature at 275°C. Orbitrap mass analyzer was used for MS1 scan with resolution set at 60,000. MS mass range was 300–1800 m/z. AGC target for each scan was set at 500,000 with maximal ion injection time set at 100 ms.

For MS2 scan, Orbitrap mass analyzer was used with an auto/normal mode at the resolution of 30,000. Only precursor ions with intensity 50,000 or higher were selected for MS2 scan. Sequence of individual MS2 scanning was from most-intense to least-intense precursor ions using a top-speed mode under time control of 4 sec. Higher energy CID (HCD) was used for fragmentation activation with 30% normalized activation energy. Quadrupole was used for precursor isolation with a 2 m/z isolation window. MS2 mass range was set auto/normal with first mass set at 110 m/z. Maximal injection time was 100 ms with AGC target set at 20,000. Ions were injected for all available parallelizable time. A 5-sec exclusion window was applied to all abundant ions to avoid repetitive MS2 scanning on the same precursor ions using 10 ppm error tolerance. Only charge states from 2 to 6 were allowed for MS2 scan, but undetermined charge states were also included. All MS2 spectra were recorded in the centroid mode.

The raw MS files were deposited to ProteomeXchange with the accession number PXD005250 and MassIVE accession number MSV000080306 (the files can be accessed using the following link: http://massive.ucsd.edu/ProteoSAFe/status.jsp?task=6a511df313a844d897459ab1eb34c07b)

The raw files were processed and analyzed using Proteome Discoverer version 2.1 (Thermo Scientific, San Jose, CA). Sequest HT search engine was used to match all MS data to a *S*. *carpocapsae* protein database (downloaded from WormBase ParaSite; steinernema_carpocapsae.PRJNA202318.WBPS5.protein.fa) and common contaminant proteins. The search parameters were following: trypsin with 2 missed cleavages, minimal peptide length for six amino acids, MS1 mass tolerance 20 ppm, MS2 mass tolerance 0.05 Da, Gln→pyro-Glu (N-term Q), oxidation (M), N-terminal acetylation as variable modifications. Only proteins with 1% FDR cut-off were considered in the final result.

### Protein domain analyses

To evaluate the prevalence of protein domains in the venom proteins and the predicted whole proteome of *S*. *carpocapsae*, we used the hmmscan program of the HMMER (3.0) software package as previously described [68], which implements probabilistic profile hidden Markov models [69]. We set our threshold *E*-value criterion at 10^−6^, so that no known false-positive matches would be detected in assigning Pfam domain identities. We ran the analysis on the predicted proteome of *S*. *carpocapsae* (WormBase ParaSite; steinernema_carpocapsae.PRJNA202318.WBPS5.protein.fa) and on the secreted venom proteins identified using mass spectrometry.

### Analyses of proteases and protease inhibitors

The sequences of all 472 protein sequences that were identified in the secreted protein products were analyzed using the MEROPS peptidase database (merops.sanger.ac.uk) [70]. A breakdown of the proteases is shown in Fig 6C.

### Gene tree analysis

Shk domain-containing proteins found in the *S*. *carpocapsae* secretome were further explored by constructing a gene tree using whole protein sequences. To do these analyses we included the 14 Shk domain-containing proteins in the secretome as well as some of the orthologs of those proteins, identified in parasite.wormbase.org. These protein sequences were aligned using MUSCLE [71]. Aligned protein sequences were then evaluated by distance analysis using the JTT matrix and a subsequent Neighbor-joining tree was created using the PHYLIP software package version 3.68, using the protdist and neighbor programs, and seqboot to calculate 1000 replicates of bootstrap analyses [72].

### Single nematode transcriptome sequencing

For *in vivo* activation of IJs, 10 waxworms were infected by 10,000 IJs in an infection Petri dish as described above. After 0.5 hr of infection, the waxworms were transferred to a new Petri dish so that the infection was relatively synchronized. After 9 hr, 12 hr and 15 hr incubation at 25°C in dark, the waxworms were dissected in 0.8% NaCl solution. The released nematodes were separated from large tissue debris and washed 3 times with 0.8% NaCl solution in a 15 ml tube. The nematode suspension was placed in a Petri dish and the nematodes (observed with a 10x6 magnification of a Leica M80 dissecting microscope with transmitted light) were individually transferred by a 10 μl pipette to another Petri dish containing 0.8% NaCl solution. This step was repeated two more times to minimize contaminating host debris.

For *in vitro* activation of IJs, 1000 IJs were incubated in 0.085 g of sponge soaked with 1 ml of 25% waxworm homogenate and 1x antibiotics (Sigma, P4083-100ML). After 12 hr incubation at 25°C in dark, nematodes were washed off the sponge foam and washed in the same way as the *in vivo* activated nematodes.

The activated nematodes were individually pipetted onto a glass slide without a coverslip and examined under a compound microscope to confirm that they have fully expanded pharyngeal bulbs (fully activated). A single fully activated nematode was pipetted in a volume of 0.5 μl onto the side wall of a clear 0.2 ml PCR tube containing 2 μl of Lysis buffer [10 mM Tris-HCl (pH 7.5–8.0), 1% Triton X-100, 1% Tween 20, 0.5 mM EDTA, 3 units of RNase inhibitor (Takara 2313A), 2 mg/ml Proteinase K (VWR, 97062–670)] at the bottom. The PCR tube was gently placed on its side on the stage of the Leica M80 dissecting microscope and the nematode was cut into 3–4 fragments using a sterile insulin syring needle (BD, 305109). The fragments were immediately spun down into the lysis buffer using a mini centrifuge. The tube was placed on ice for 30–60 min. 8 nematodes were individually processed for each condition, i.e., non-activated, *in vitro* activated for 12h, *in vivo* activated for 9 hr, 12 hr, and 15 hr.

Library preparation was done using the Illumina Nextera tagmentation protocol [73, 74]. Input DNA and reagents were scaled down so that 20ng of cDNA (2.5 ng/ uL) was combined with 12 uL of tagment buffer and 2.1 uL of transposase enzyme. The tagmentation reaction was carried out for 5 min at 55°C in a thermocycler (lid heated to 105°C). The tagmented DNA was cleaned using a Qiagen DNA purification kit, and eluted from the Qiagen column with 30 uL of EB. The sample was mixed together with 35 μL of Phusion high fidelity master mix, 2.5 μL of 25 μM Nextera adapter ID, and 2.5 μL of 25 μM Nextera adapter Ad_noMX in a PCR tube. Samples were spun down quickly, and amplified for 6 cycles using the PCR program with the following settings: 72°C for 5 min, 98°C for 30 s, [98°C for 10 s, 63°C for 30 s, 72°C for 1 min] for 6 cycles, 72°C for 5 min, and hold at 4°C.

PCR primers were cleaned from the sample by mixing the sample with a 1:1 ratio of Ampure XP beads. Samples were placed on a magnetic stand, and washed with three 200 uL washes of 80% ethanol. The beads were dried for 5 min and the library was eluted from the beads in 30 uL of elution buffer. Library concentrations were measured with the Qubit fluorometer and Bioanalyzed with the 2100 Agilent Bioanalyzer.

Sample libraries had an average fragment size of 450bp and were sequenced as paired-end 43 bp on the Illumina NextSeq 500 to an average depth of 20 million reads. Reads for single worm RNA-seq samples were submitted to Gene Expression Omnibus (GEO) under the accession number GSE89961 (https://www.ncbi.nlm.nih.gov/geo/query/acc.cgi?token=qdcbequejxcfzef&acc=GSE89961).

### Gene expression quantification

The *S*. *carpocapsae* transcriptome (PRJNA202318 downloaded from WormBase ParaSite version WS254) was prepared for indexing using the RSEM command (version 1.2.12) rsem-prepare-reference [68, 75]. The unstranded, paired-end 43 bp RNA-seq reads were mapped to the transcriptome using Bowtie 0.12.8 with the following options: -X 1500 -a -m 200 -S—seedlen 25 -n 2—offrate 1 -p 64 -v 3 [76]. Gene expression was quantified with RSEM using the command rsem-calculate-expression. RNA-seq reads were also mapped to the genome using Tophat [77] to compare read mapping percentages between the genome and transcriptome.

### Batch correction and normalization of gene expression data

Gene expression in transcripts per million (TPM) from RSEM were batch corrected with the R package limma to normalize for technical differences since the 9 hr and 12 hr *in vivo* activated samples were collected and processed on a different day [78] from other samples. Gene expression counts was quantile normalized using normalizeQuantiles in R.

### Differential gene expression analysis

Differential gene expression between activation stages was determined using EdgeR [79]. TPMs were normalized by library size with calcNormFactors(d). For all differential expression (DE) analyses, genes were called as differentially expressed if they had FDRs < 0.05 and fold changes > 2X.

### Gene Ontology enrichment analyses

Fisher’s exact tests were performed on sets of genes that were differentially expressed according to edgeR or dynamically expressed according to maSigPro using Blast2GO [80]. Genes that are enriched in GO categories with FDRs < 0.05 were considered statistically significant.

### Assessing differential gene expression dynamics during the *in vivo* activation time course with maSigPro

maSigPro was run as a single time series to assess the gene expression dynamics during *in vivo* activation time course for all genes (28,313) and for the 472 venom genes. TPMs were normalized using calcNormFactors() in R, and DE genes were called with FDR < 0.01.

### Venom protein conservation across nematode parasites

Venom protein orthology relationships were determined across parasite proteomes (downloaded from parasite.wormbase.org) using OrthoMCL version 1.4 for the following parasitic nematodes: *S*. *carpocapsae* (PRJNA202318. WBPS8), *S*. *stercoralis* (PRJEB528.WBPS8), *T*. *canis* (PRJNA248777.WBPS8), *B*. *malayi* (PRJNA10729.WBPS8), *H*. *contortus* (PRJNA205202.WBPS8), *A*. *suum* (PRJNA80881.WBPS8), *O*. *dentatum* (PRJNA72579.WBPS8), *A*. *ceylanicum* (PRJNA231479.WBPS8), *D*. *viviparous* (PRJEB5116.WBPS8), *H*. *bacteriophora*(PRJNA13977.WBPS8), *S*. *feltiae* (PRJNA204661.WBPS8), *S*. *glaseri* (PRJNA204943.WBPS8), *S*. *monticolum* (PRJNA205067.WBPS8), *S*. *scapterisci* (PRJNA204942.WBPS8). Prior to clustering proteins in OrthoMCL, proteomes for each species were filtered to retain only the longest proteins corresponding to each gene sequence. OrthoMCL was run with the default settings (blast p-value cutoff = 1e-5, MCL inflation = 1.5). A custom script was run to reformat the results of the OrthoMCL orthology clustering into the venom protein conservation table. Essentially, the script counts the number of proteins from each parasite that clusters with the secreted venom proteins, and categorizes the clusters into whether they are *Steinernema-*conserved venom protein clusters, vertebrate-conserved venom protein clusters (meaning that at least one vertebrate parasitic protein clusters with an *S*. *carpocapsae* venom protein), or *S*. *carpocapsae*-only protein clusters. It also calculates and ranks the contributions each orthology cluster has to the fraction of venom protein molecules in the secretion (“Fraction of venom” and “Rank” column in the venom conservation table).

### Gene expression analysis of *S*. *stercoralis* venom protein orthologs

Previously published infective juvenile (ERR146948 and ERR146949) and activated infective juvenile (ERR146945 and ERR146946) RNA-seq data sets for *S*. *stercoralis* were obtained from: http://www.ebi.ac.uk/arrayexpress/experiments/E-MTAB-1164/ [54]. Messenger RNA transcripts sequences (PRJEB528.WBPS8) were downloaded from Wormbase Parasite. The transcriptome sequences were indexed for bowtie and rsem with rsem-prepare-reference using rsem version 1.2.31 [75]. Reads were mapped to the indexed transcriptome with bowtie version 1.0.0 with the following settings: -X 1500 -m 200 -S—seedlen 25 -n 2—trim3 3—offrate 1 -v 3 [76]. Gene expression was quantified with rsem. Transcripts per million were used for the downstream analyses.

## Supporting information

S1 Fig Toxicity tests of venom of activated axenic IJs. (A) Survival of flies within 72 hrs after injection of 10 and 20 ng of venom proteins of axenic IJs. (B) Survival of flies injected by 10 and 20 ng of venom proteins of axenic IJs until all flies died. (C) Survival of flies injected by 20 ng of venom proteins harvested from axenic IJs that were activated for different amounts of time in waxworm homogenate.
(TIF)

S2 Fig Culturing of axenic *S*. *carpocapsae* IJs on colonization defective mutant bacteria of *Xenorhabdus nematophila*. (A) Colonization defective mutant bacteria of *X*.*nematophila* on NBTA (left) and MacConkey (right) plates. (B) Surface-sterilized eggs/embryos from gravid females. (C) Nematodes developed from eggs/embryos feeding on mutant bacteria. (D) Bacterial test of symbiotic IJs. (E) Bacterial test of axenic IJs. Three controls were included in (D) and (E): (1) 50 μl of the supernatant of the washed surface-sterilized IJs to validate the completeness of surface sterilization; (2) 50 μl of autoclaved 0.8% NaCl solution to confirm that the original solution for washing IJs was sterile; and (3) 50 μl of 0.8% NaCl solution rinsed with an autoclaved tissue grinder to show that the grinder was sterile. The quarter 4 was 50 μl homogenate of 1000 surface-sterilized IJs.
(TIF)

S3 Fig Injected waxworm larvae. (A) Photograph of last instar waxworm larvae 5 days after injection with PBS. (B) Photograph of last instar waxworm larvae 5 days after injection with 4 μg of venom collected from axenic *S*. *carpocapsae* IJs that had been activated for 12 h in waxworm homogenate. (C) Another photograph of last instar waxworm larvae 5 days after injection with 4 μg of venom collected from axenic *S*. *carpocapsae* IJs that had been activated for 12 h in waxworm homogenate.
(TIF)

S4 Fig Individual nematode RNA-seq sample statistics. (A) Plot showing the percentage of reads mapped to the *S*. *carpocapsae* transcriptome and genome for each individual IJ RNA-seq sample. Samples are grouped based on their activation stages or methods of activation. (B) Boxplot showing the numbers of genes expressed greater than 5 counts for each activation stage and method of activation.
(TIF)

S5 Fig Bar charts showing the GO terms that were significantly enriched (Fisher’s exact test, FDR < 0.05) in the genes represented by the cluster 1, 2, 3, and 4 maSigPro plots in Fig 3D. (TIF)

S6 Fig Comparison of transcriptomes of IJs activated with different methods and time. (A) Comparison of the transcriptome of 12h *in vitro* activated IJs with that of 9h *in vivo* activated IJs. (B) Comparison of the transcriptome of 12h *in vitro* activated IJs with that of 12h *in vivo* activated IJs.
(TIF)

S7 Fig Bar charts showing the GO terms that were significantly enriched (Fisher’s exact test, FDR < 0.05) in the genes represented by the categories in Fig 4C. (TIF)

S8 Fig Correlation between RNA and protein levels of the top 100 venom proteins (abundance based on emPAI values). A few examples of proteins with different types of RNA-Protein correlations are shown on the right. g21515; g23482 and g19074 have good RNA-Protein correlation; g1859 and g1800 have high protein and low RNA levels; g1988; g15816 and g13216 are examples for those with high RNA and low protein levels.
(TIF)

S9 Fig Gene expression of 168 venom protein orthologs in *S*. *stercoralis*. A bar graph showing the expression levels of 168 venom protein orthologs in *S*. *stercoralis*. Blue bars shows the expression of these genes in the infectious 3^rd^ larval stage, equivalent to *S*. *carpocapsae* IJs. Red bars show the expression of these genes in tissue migrating 3^rd^ larval stage, equivalent to activated or activating *S*. *carpocapsae* IJs. RNA-seq data came from previously published work [54].
(TIF)

S1 Table The percentage of exsheathed IJs without activation. (DOCX)

S2 Table *S*. *carpocapsae* IJ time course activation rates. (DOCX)

S3 Table Estimated venom amount per IJ and the number of IJs required for secreting enough venom (10 ng) to kill Drosophila. (DOCX)

S4 Table A list of 472 *S*. *carpocapsae* venom proteins identified by mass spectrometry (FDR<1%). (XLSX)

S5 Table A table showing the number of orthologous clusters that contain at least one *S*. *carpocapsae* venom protein and at least one protein from the listed species. (DOCX)

S1 Video A video of a non-activated *S*. *carpocapsae* IJ (10 x 40 magnification). (M4V)

S2 Video A video of a partially activated *S*. *carpocapsae* IJ (10 x 40 magnification). (M4V)

S3 Video A video of a fully activated *S*. *carpocapsae* IJ (10 x 40 magnification). (M4V)

S4 Video A video of last instar waxworm larvae injected with PBS. The video was filmed 24 hours after the injection.
(MP4)

S5 Video A video of last instar waxworm larvae injected with 4 μg of venom proteins from axenic *S*. *carpocapsae* that had been activated in waxworm extract for 12 h. The video was filmed 24 hours after the injection.
(MP4)

S6 Video A video of last instar waxworm larvae injected with PBS. The video was filmed 5 days after the injection.
(MP4)

S7 Video A video of last instar waxworm larvae injected with 4 μg of venom proteins from axenic *S*. *carpocapsae* that had been activated in waxworm extract for 12 h. The video was filmed 5 days after the injection.
(MP4)

## References

[1] Hotez PJ, Alvarado M, Basanez MG, Bolliger I, Bourne R, Boussinesq M, et al The global burden of disease study 2010: interpretation and implications for the neglected tropical diseases. PLoS Negl Trop Dis. 2014;8(7):e2865 PubMed Central PMCID: PMCPMC4109880. doi: 10.1371/journal.pntd.0002865 25058013 10.1371/journal.pntd.0002865 PMC4109880

[2] Pullan RL, Smith JL, Jasrasaria R, Brooker SJ. Global numbers of infection and disease burden of soil transmitted helminth infections in 2010. Parasit Vectors. 2014;7:37 PubMed Central PMCID: PMCPMC3905661. doi: 10.1186/1756-3305-7-37 24447578 10.1186/1756-3305-7-37 PMC3905661

[3] Hewitson JP, Maizels RM. Vaccination against helminth parasite infections. Expert Rev Vaccines. 2014;13(4):473–87. doi: 10.1586/14760584.2014.893195 24606541 10.1586/14760584.2014.893195

[4] Shepherd C, Navarro S, Wangchuk P, Wilson D, Daly NL, Loukas A. Identifying the immunomodulatory components of helminths. Parasite Immunol. 2015;37(6):293–303. doi: 10.1111/pim.12192 25854639 10.1111/pim.12192

[5] Hotez PJ, Diemert D, Bacon KM, Beaumier C, Bethony JM, Bottazzi ME, et al The human hookworm vaccine. Vaccine. 2013;31 Suppl 2:B227–32. PubMed Central PMCID: PMCPMC3988917. 23598487 10.1016/j.vaccine.2012.11.034 PMC3988917

[6] Navarro S, Pickering DA, Ferreira IB, Jones L, Ryan S, Troy S, et al Hookworm recombinant protein promotes regulatory T cell responses that suppress experimental asthma. Science Translational Medicine. 2016;8(362):362ra143 doi: 10.1126/scitranslmed.aaf8807 27797959 10.1126/scitranslmed.aaf8807

[7] Ward JD. Rendering the intractable more tractable: Tools from *Caenorhabditis elegans* ripe for import into parasitic nematodes. Genetics. 2015;201(4):1279–94. PubMed Central PMCID: PMCPMC4676526. doi: 10.1534/genetics.115.182717 26644478 10.1534/genetics.115.182717 PMC4676526

[8] Blaxter M, Koutsovoulos G. The evolution of parasitism in Nematoda. Parasitology. 2015;142:S26–S39. doi: 10.1017/S0031182014000791 24963797 10.1017/S0031182014000791 PMC4413787

[9] Castelletto ML, Gang SS, Okubo RP, Tselikova AA, Nolan TJ, Platzer EG, et al Diverse host-seeking behaviors of skin-penetrating nematodes. PLoS Pathogens. 2014;10(8):e1004305 PubMed Central PMCID: PMC4133384. doi: 10.1371/journal.ppat.1004305 25121736 10.1371/journal.ppat.1004305 PMC4133384

[10] Lee JH, Dillman AR, Hallem EA. Temperature-dependent changes in the host-seeking behaviors of parasitic nematodes. BMC biology. 2016;14. 10.1186/s12915-016-0259-0 PMC4858831 27154502

[11] Dillman AR, Chaston JM, Adams BJ, Ciche TA, Goodrich-Blair H, Stock SP, et al An entomopathogenic nematode by any other name. PLoS Pathogens. 2012;8(3):e1002527 doi: 10.1371/journal.ppat.1002527 22396642 10.1371/journal.ppat.1002527 PMC3291613

[12] Kaya HK, Gaugler R. Entomopathogenic nematodes. Annu Rev Entomol. 1993;38:181–206.

[13] Lewis EE, Clarke DJ. Nematode parasites and entomopathogens In: Vega FE, Kaya HK, editors. Insect Pathology. 2nd ed: Elsevier; 2012 p. 395–424.

[14] Goodrich-Blair H. They've got a ticket to ride: *Xenorhabdus nematophila*-*Steinernema carpocapsae* symbiosis. Curr Opin Microbiol. 2007;10(3):225–30. doi: 10.1016/j.mib.2007.05.006 17553732 10.1016/j.mib.2007.05.006

[15] Goodrich-Blair H, Clarke DJ. Mutualism and pathogenesis in *Xenorhabdus* and *Photorhabdus*: two roads to the same destination. Molecular Microbiology. 2007;64(2):260–8. doi: 10.1111/j.1365-2958.2007.05671.x 17493120 10.1111/j.1365-2958.2007.05671.x

[16] Han R, Ehlers RU. Pathogenicity, development, and reproduction of *Heterorhabditis bacteriophora* and *Steinernema carpocapsae* under axenic *in vivo* conditions. J Invertebr Pathol. 2000;75:55–8. doi: 10.1006/jipa.1999.4900 10631058 10.1006/jipa.1999.4900

[17] Poinar GO Jr., Thomas GM. Significance of *Achromobacter nematophilus* Poinar and Thomas (Achromobacteraceae: Eubacteriales) in the development of the nematode, DD-136 (Neoaplectana sp. Steinernematidae). Parasitology. 1966;56(2):385–90. Epub 1966/05/01. 4960247 10.1017/s0031182000070980

[18] Sicard M, Le Brun N, Pages S, Godelle B, Boemare N, Moulia C. Effect of native *Xenorhabdus* on the fitness of their *Steinernema* hosts: Contrasting types of interaction. Parasitology Research. 2003;91(6):520–4. doi: 10.1007/s00436-003-0998-z 14557877 10.1007/s00436-003-0998-z

[19] Burman M. *Neoaplectana carpocapsae*—toxin production by axenic insect parasitic nematodes. Nematologica. 1982;28(1):62–70.

[20] Walter TN, Dunphy GB, Mandato CA. *Steinernema carpocapsae* DD136: Metabolites limit the non-self adhesion responses of haemocytes of two lepidopteran larvae, *Galleria mellonella* (F. Pyralidae) and *Malacosoma disstria* (F. Lasiocampidae). Exp Parasitol. 2008;120(2):161–74. doi: 10.1016/j.exppara.2008.07.001 18656470 10.1016/j.exppara.2008.07.001

[21] Pye AE, Burman M. *Neoaplectana carpocapsae*—infection and reproduction in large pine weevil larvae, *Hylobius abietis*. Exp Parasitol. 1978;46(1):1–11. 729687 10.1016/0014-4894(78)90151-0

[22] Martens EC, Goodrich-Blair H. The *Steinernema carpocapsae* intestinal vesicle contains a subcellular structure with which *Xenorhabdus nematophila* associates during colonization initiation. Cellular microbiology. 2005;7(12):1723–35. doi: 10.1111/j.1462-5822.2005.00585.x 16309459 10.1111/j.1462-5822.2005.00585.x

[23] Balasubramanian N, Hao YJ, Toubarro D, Nascimento G, Simoes N. Purification, biochemical and molecular analysis of a chymotrypsin protease with prophenoloxidase suppression activity from the entomopathogenic nematode *Steinernema carpocapsae*. Int J Parasit. 2009;39(9):975–84. 10.1016/j.ijpara.2009.01.012 19249304

[24] Balasubramanian N, Nascimento G, Ferreira R, Martinez M, Simoes N. Pepsin-like aspartic protease (Sc-ASP155) cloning, molecular characterization and gene expression analysis in developmental stages of nematode *Steinernema carpocapsae*. Gene. 2012;500(2):164–71. doi: 10.1016/j.gene.2012.03.062 22503896 10.1016/j.gene.2012.03.062

[25] Balasubramanian N, Toubarro D, Nascimento G, Ferreira R, Simoes N. Purification, molecular characterization and gene expression analysis of an aspartic protease (Sc-ASP113) from the nematode *Steinernema carpocapsae* during the parasitic stage. Molecular & Biochemical Parasitology. 2012;182(1–2):37–44. 22178695 10.1016/j.molbiopara.2011.12.001

[26] Balasubramanian N, Toubarro D, Simoes N. Biochemical study and in vitro insect immune suppression by a trypsin-like secreted protease from the nematode *Steinernema carpocapsae*. Parasite Immunol. 2010;32(3):165–75. doi: 10.1111/j.1365-3024.2009.01172.x 20398179 10.1111/j.1365-3024.2009.01172.x

[27] Hao YJ, Montiel R, Nascimento G, Toubarro D, Simoes N. Identification and expression analysis of the *Steinernema carpocapsae* elastase-like serine protease gene during the parasitic stage. Exp Parasitol. 2009;122(1):51–60. doi: 10.1016/j.exppara.2009.01.014 19545520 10.1016/j.exppara.2009.01.014

[28] Jing Y, Toubarro D, Hao Y, Simoes N. Cloning, characterisation and heterologous expression of an astacin metalloprotease, Sc-AST, from the entomoparasitic nematode *Steinernema carpocapsae*. Molecular & Biochemical Parasitology. 2010;174(2):101–8. 10.1016/j.molbiopara.2010.07.004 20670659

[29] Toubarro D, Avila MM, Hao YJ, Balasubramanian N, Jing YJ, Montiel R, et al A serpin released by an entomopathogen impairs clot formation in insect defense system. Plos One. 2013;8(7):e69161 doi: 10.1371/journal.pone.0069161 23874900 10.1371/journal.pone.0069161 PMC3712955

[30] Toubarro D, Avila MM, Montiel R, Simoes N. A pathogenic nematode targets recognition proteins to avoid insect defenses. Plos One. 2013;8(9):e75691 PubMed Central PMCID: PMC3787073. doi: 10.1371/journal.pone.0075691 24098715 10.1371/journal.pone.0075691 PMC3787073

[31] Toubarro D, Lucena-Robles M, Nascimento G, Costa G, Montiel R, Coelho AV, et al An apoptosis-inducing serine protease secreted by the entomopathogenic nematode S*teinernema carpocapsae*. Int J Parasit. 2009;39(12):1319–30. 10.1016/j.ijpara.2009.04.013 19481087

[32] Toubarro D, Lucena-Robles M, Nascimento G, Santos R, Montiel R, Verissimo P, et al Serine protease-mediated host invasion by the parasitic nematode *Steinernema carpocapsae*. J Biol Chem. 2010;285(40):30666–75. doi: 10.1074/jbc.M110.129346 20656686 10.1074/jbc.M110.129346 PMC2945561

[33] Lee D. The physiology of nematodes. San Francisco: W. H. Freeman and Company; 1965.

[34] Rogers WP, Petronijevic T. The infective stage and the development of nematodes In: Symons LEA, Donald AD, Dineen JK, editors. Biology and control of endoparasites. Sydney: Academic Press; 1982 p. 3–28.

[35] Bonner TP. Initiation of development *in vitro* of 3rd-stage *Nippostrongylus brasiliensis*. Journal of Parasitology. 1979;65(1):74–8. 448602

[36] Hawdon JM, Volk SW, Pritchard DI, Schad GA. Resumption of feeding *in vitro* by hookworm 3rd-stage larvae—a comparative study. Journal of Parasitology. 1992;78(6):1036–40. 1491295

[37] Bonner TP. Changes in the structure of *Nippostrongylus brasiliensis* intestinal cells during development from the free-living to the parasitic stages. Journal of Parasitology. 1979;65(5):745–50. 512766

[38] Hawdon JM, Schad GA. Serum-stimulated feeding *in vitro* by 3rd-stage infective larvae of the canine hookworm *Ancylostoma caninum*. Journal of Parasitology. 1990;76(3):394–8. 2112598

[39] Hawdon JM, Schad GA. Albumin and a dialyzable serum factor stimulate feeding *in vitro* by 3rd-stage larvae of the canine hookworm *Ancylostoma caninum*. Journal of Parasitology. 1991;77(4):587–91. 1713962

[40] Petronijevic T, Rogers WP. Gene activity and the development of early parasitic stages of nematodes. Int J Parasit. 1983;13(2):197–9. 10.1016/0020-7519(83)90012-7 6853019

[41] Hawdon JM, Jones BF, Perregaux MA, Hotez PJ. *Ancylostoma caninum*—Metalloprotease release coincides with activation of infective larvae *in vitro*. Exp Parasitol. 1995;80(2):205–11. doi: 10.1006/expr.1995.1025 7895832 10.1006/expr.1995.1025

[42] Crook M. The dauer hypothesis and the evolution of parasitism: 20 years on and still going strong. Int J Parasit. 2014;44(1):1–8. 10.1016/j.ijpara.2013.08.004 PMC3947200 24095839

[43] Moshayov A, Koltai H, Glazer I. Molecular characterisation of the recovery process in the entomopathogenic nematode *Heterorhabditis bacteriophora*. Int J Parasit. 2013;43(10):843–52. 10.1016/j.ijpara.2013.05.009 23806512

[44] Golden JW, Riddle DL. A pheromone influences larval development in the nematode *Caenorhabditis elegans*. Science. 1982;218(4572):578–80. 6896933 10.1126/science.6896933

[45] Lu D, Sepulveda C, Dillman AR. Infective juveniles of the entomopathogenic nematode *Steinernema scapterisci* are preferentially activated by cricket tissue. Plos One. 2016;Accepted. 10.1371/journal.pone.0169410 PMC5207650 28046065

[46] Loukas A, Hintz M, Linder D, Mullin NP, Parkinson J, Tetteh KK, et al A family of secreted mucins from the parasitic nematode *Toxocara canis* bears diverse mucin domains but shares similar flanking six-cysteine repeat motifs. J Biol Chem. 2000;275(50):39600–7. doi: 10.1074/jbc.M005632200 10950959 10.1074/jbc.M005632200

[47] Allen JE, MacDonald AS. Profound suppression of cellular proliferation mediated by the secretions of nematodes. Parasite Immunol. 1998;20(5):241–7. Epub 1998/07/04. 9651925 10.1046/j.1365-3024.1998.00151.x

[48] McDermott L, Cooper A, Kennedy MW. Novel classes of fatty acid and retinol binding protein from nematodes. Mol Cell Biochem. 1999;192(1–2):69–75. 10331660

[49] Castillo JC, Shokal U, Eleftherianos I. Immune gene transcription in *Drosophila* adult flies infected by entomopathogenic nematodes and their mutualistic bacteria. J Insect Physiol. 2013;59(2):179–85. doi: 10.1016/j.jinsphys.2012.08.003 22902989 10.1016/j.jinsphys.2012.08.003

[50] Hallem EA, Rengarajan M, Ciche TA, Sternberg PW. Nematodes, bacteria, and flies: a tripartite model for nematode parasitism. Current biology: CB. 2007;17(10):898–904. doi: 10.1016/j.cub.2007.04.027 17475494 10.1016/j.cub.2007.04.027

[51] Heungens K, Cowles CE, Goodrich-Blair H. Identification of *Xenorhabdus nematophila* genes required for mutualistic colonization of S*teinernema carpocapsae* nematodes. Molecular Microbiology. 2002;45(5):1337–53. 12207701 10.1046/j.1365-2958.2002.03100.x

[52] Hao YJ, Montiel R, Abubucker S, Mitreva M, Simoes N. Transcripts analysis of the entomopathogenic nematode *Steinernema carpocapsae* induced in vitro with insect haemolymph. Molecular and biochemical parasitology. 2010;169(2):79–86. doi: 10.1016/j.molbiopara.2009.10.002 19836423 10.1016/j.molbiopara.2009.10.002 PMC4010113

[53] Zaslaver A, Baugh LR, Sternberg PW. Metazoan operons accelerate recovery from growth-arrested states. Cell. 2011;145(6):981–92. doi: 10.1016/j.cell.2011.05.013 21663799 10.1016/j.cell.2011.05.013 PMC3152313

[54] Stoltzfus JD, Minot S, Berriman M, Nolan TJ, Lok JB. RNAseq analysis of the parasitic nematode Strongyloides stercoralis reveals divergent regulation of canonical dauer pathways. PLoS Negl Trop Dis. 2012;6(10):e1854 PubMed Central PMCID: PMCPMC3493385. doi: 10.1371/journal.pntd.0001854 23145190 10.1371/journal.pntd.0001854 PMC3493385

[55] Lewis EE, Grewal PS, Gauger R. Hierarchical order of host cues in parasite foraging strategies. Parasitology. 1995;110:207–13.

[56] Buck AH, Coakley G, Simbari F, McSorley HJ, Quintana JF, Le Bihan T, et al Exosomes secreted by nematode parasites transfer small RNAs to mammalian cells and modulate innate immunity. Nat Commun. 2014;5. 10.1038/ncomms6488 PMC4263141 25421927

[57] Choe A, von Reuss SH, Kogan D, Gasser RB, Platzer EG, Schroeder FC, et al Ascaroside signaling is widely conserved among nematodes. Current Biology. 2012;22(9):772–80. doi: 10.1016/j.cub.2012.03.024 22503501 10.1016/j.cub.2012.03.024 PMC3360977

[58] Croese J, Giacomin P, Navarro S, Clouston A, McCann L, Dougall A, et al Experimental hookworm infection and gluten microchallenge promote tolerance in celiac disease. J Allergy Clin Immun. 2015;135(2):508–U677. doi: 10.1016/j.jaci.2014.07.022 25248819 10.1016/j.jaci.2014.07.022

[59] von Reuss SH, Schroeder FC. Combinatorial chemistry in nematodes: Modular assembly of primary metabolism-derived building blocks. Natural Product Reports. 2015;32(7):994–1006. doi: 10.1039/c5np00042d 26059053 10.1039/c5np00042d PMC4884655

[60] Borloo J, De Graef J, Peelaers I, Nguyen DL, Mitreva M, Devreese B, et al In-depth proteomic and glycomic analysis of the adult-stage *Cooperia oncophora* excretome/secretome. Journal of Proteome Research. 2013;12(9):3900–11. PubMed Central PMCID: PMCPMC3883574. doi: 10.1021/pr400114y 23895670 10.1021/pr400114y PMC3883574

[61] Soblik H, Younis AE, Mitreva M, Renard BY, Kirchner M, Geisinger F, et al Life cycle stage-resolved proteomic analysis of the excretome/secretome from *Strongyloides ratti*—identification of stage-specific proteases. Molecular & Cellular Proteomics. 2011;10(12):M111 010157. PubMed Central PMCID: PMCPMC3237078. 10.1074/mcp.M111.010157 PMC3237078 21964353

[62] Sotillo J, Sanchez-Flores A, Cantacessi C, Harcus Y, Pickering D, Bouchery T, et al Secreted proteomes of different developmental stages of the gastrointestinal nematode *Nippostrongylus brasiliensis*. Molecular & Cellular Proteomics. 2014;13(10):2736–51. PubMed Central PMCID: PMCPMC4188999. 24994561 10.1074/mcp.M114.038950 PMC4188999

[63] Pham LN, Dionne MS, Shirasu-Hiza M, Schneider DS. A specific primed immune response in *Drosophila* is dependent on phagocytes. PLoS Pathog. 2007;3(3):e26 PubMed Central PMCID: PMC1817657. doi: 10.1371/journal.ppat.0030026 17352533 10.1371/journal.ppat.0030026 PMC1817657

[64] White GF. A method for obtaining infective nematode larvae from cultures. Science. 1927;66(1709):302–3. 10.1126/science.66.1709.302-a 17749713

[65] Ragsdale EJ, Mundo-Ocampo M, Bumbarger DJ, Baldwin JG. *Cervidellus sonorensis* n. sp. (Nematoda: Cephalobidae) from the desert of Anza-Borrego, CA, USA. Nematology. 2011;13:607–17.

[66] Blackburn D, Wood PL, Burk TJ, Crawford B, Wright SM, Adams BJ. Evolution of virulence in Photorhabdus spp., entomopathogenicnematode symbionts. Systematic and Applied Microbiology. 2016;39:173–9. doi: 10.1016/j.syapm.2016.02.003 27020955 10.1016/j.syapm.2016.02.003

[67] Drakakaki G, van de Ven W, Pan S, Miao Y, Wang J, Keinath NF, et al Isolation and proteomic analysis of the SYP61 compartment reveal its role in exocytic trafficking in *Arabidopsis*. Cell Research. 2012;22(2):413–24. PubMed Central PMCID: PMCPMC3271593. doi: 10.1038/cr.2011.129 21826108 10.1038/cr.2011.129 PMC3271593

[68] Dillman AR, Macchietto M, Porter CF, Rogers A, Williams B, Antoshechkin I, et al Comparative genomics of *Steinernema* reveals deeply conserved gene regulatory networks. Genome Biology. 2015;16:200 doi: 10.1186/s13059-015-0746-6 26392177 10.1186/s13059-015-0746-6 PMC4578762

[69] Finn RD, Clements J, Eddy SR. HMMER web server: interactive sequence similarity searching. Nucleic Acids Res. 2011;39:W29–37. PubMed Central PMCID: PMC3125773. doi: 10.1093/nar/gkr367 21593126 10.1093/nar/gkr367 PMC3125773

[70] Rawlings ND, Barrett AJ, Bateman A. MEROPS: the database of proteolytic enzymes, their substrates and inhibitors. Nucleic Acids Res. 2012;40:D343–50. Epub 2011/11/17. PubMed Central PMCID: PMC3245014. doi: 10.1093/nar/gkr987 22086950 10.1093/nar/gkr987 PMC3245014

[71] Edgar RC. MUSCLE: a multiple sequence alignment method with reduced time and space complexity. Bmc Bioinformatics. 2004;5:113 doi: 10.1186/1471-2105-5-113 15318951 10.1186/1471-2105-5-113 PMC517706

[72] Felsenstein J. PHYLIP (Phylogeny Inference Package). 3.6 ed2005.

[73] Gertz J, Varley KE, Davis NS, Baas BJ, Goryshin IY, Vaidyanathan R, et al Transposase mediated construction of RNA-seq libraries. Genome Res. 2012;22(1):134–41. PubMed Central PMCID: PMCPMC3246200. doi: 10.1101/gr.127373.111 22128135 10.1101/gr.127373.111 PMC3246200

[74] Picelli S, Faridani OR, Bjorklund AK, Winberg G, Sagasser S, Sandberg R. Full-length RNA-seq from single cells using Smart-seq2. Nature protocols. 2014;9(1):171–81. doi: 10.1038/nprot.2014.006 24385147 10.1038/nprot.2014.006

[75] Li B, Dewey CN. RSEM: Accurate transcript quantification from RNA-Seq data with or without a reference genome. Bmc Bioinformatics. 2011;12. 10.1186/1471-2105-12-323 PMC3163565 21816040

[76] Langmead B, Trapnell C, Pop M, Salzberg SL. Ultrafast and memory-efficient alignment of short DNA sequences to the human genome. Genome Biol. 2009;10(3):R25 PubMed Central PMCID: PMC2690996. doi: 10.1186/gb-2009-10-3-r25 19261174 10.1186/gb-2009-10-3-r25 PMC2690996

[77] Trapnell C, Pachter L, Salzberg SL. TopHat: discovering splice junctions with RNA-Seq. Bioinformatics. 2009;25(9):1105–11. doi: 10.1093/bioinformatics/btp120 19289445 10.1093/bioinformatics/btp120 PMC2672628

[78] Ritchie ME, Phipson B, Wu D, Hu Y, Law CW, Shi W, et al limma powers differential expression analyses for RNA-sequencing and microarray studies. Nucleic Acids Res. 2015;43(7):e47 PubMed Central PMCID: PMCPMC4402510. doi: 10.1093/nar/gkv007 25605792 10.1093/nar/gkv007 PMC4402510

[79] Robinson MD, McCarthy DJ, Smyth GK. edgeR: a Bioconductor package for differential expression analysis of digital gene expression data. Bioinformatics. 2010;26(1):139–40. PubMed Central PMCID: PMC2796818. doi: 10.1093/bioinformatics/btp616 19910308 10.1093/bioinformatics/btp616 PMC2796818

[80] Conesa A, Gotz S, Garcia-Gomez JM, Terol J, Talon M, Robles M. Blast2GO: a universal tool for annotation, visualization and analysis in functional genomics research. Bioinformatics. 2005;21(18):3674–6. doi: 10.1093/bioinformatics/bti610 16081474 10.1093/bioinformatics/bti610

