# Supplementary figures and images for: Activated entomopathogenic nematode infective juveniles release lethal venom proteins
Source: PLoS Pathog. 2017 Apr 20;13(4):e1006302. doi: 10.1371/journal.ppat.1006302 (PMC5398726; doi:10.1371/journal.ppat.1006302)

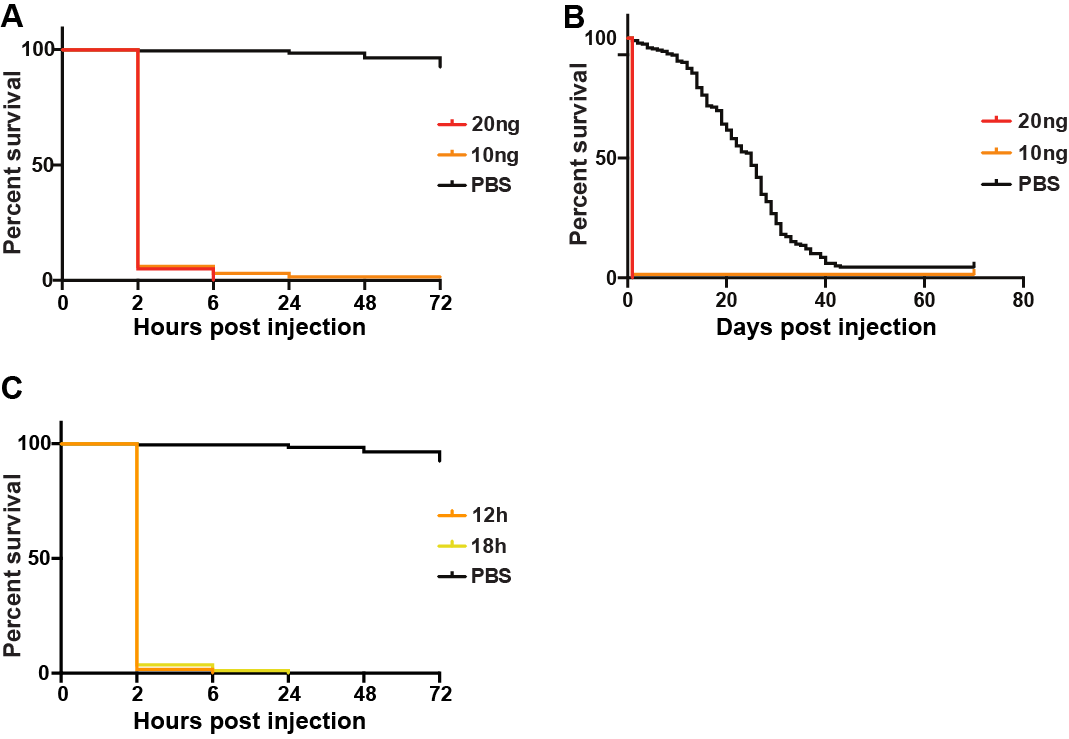

Supplement: S1 Fig — (A) Survival of flies within 72 hrs after injection of 10 and 20 ng of venom proteins of axenic IJs. (B) Survival of flies injected by 10 and 20 ng of venom proteins of axenic IJs until all flies died. (C) Survival of flies injected by 20 ng of venom proteins harvested from axenic IJs that were activated for different amounts of time in waxworm homogenate. (TIF) [file ppat.1006302.s001.tif]

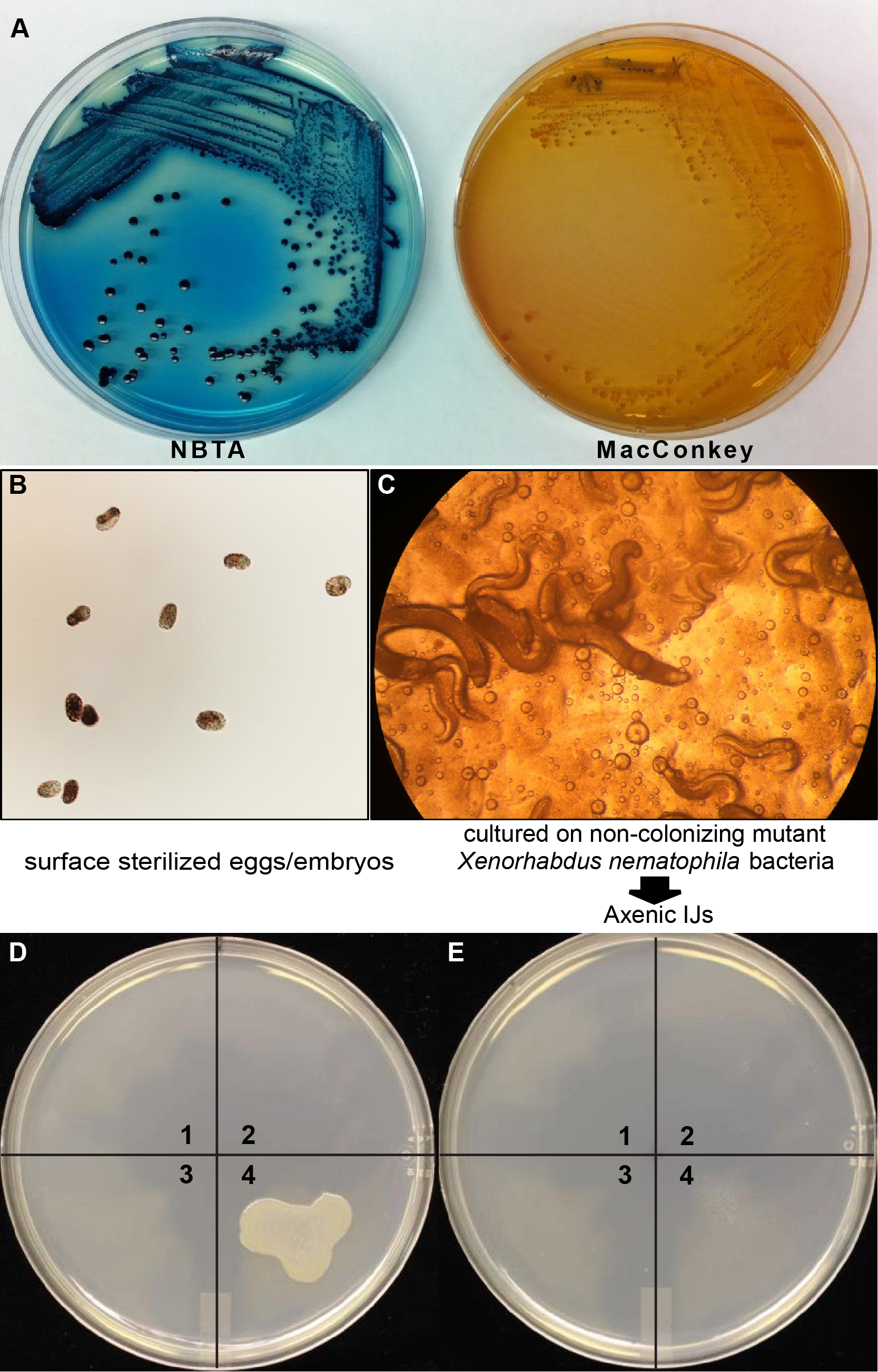

Supplement: S2 Fig — (A) Colonization defective mutant bacteria of X.nematophila on NBTA (left) and MacConkey (right) plates. (B) Surface-sterilized eggs/embryos from gravid females. (C) Nematodes developed from eggs/embryos feeding on mutant bacteria. (D) Bacterial test of symbiotic IJs. (E) Bacterial test of axenic IJs. Three controls were included in (D) and (E): (1) 50 μl of the supernatant of the washed surface-sterilized IJs to validate the completeness of surface sterilization; (2) 50 μl of autoclaved 0.8% NaCl solution to confirm that the original solution for washing IJs was sterile; and (3) 50 μl of 0.8% NaCl solution rinsed with an autoclaved tissue grinder to show that the grinder was sterile. The quarter 4 was 50 μl homogenate of 1000 surface-sterilized IJs. (TIF) [file ppat.1006302.s002.tif]

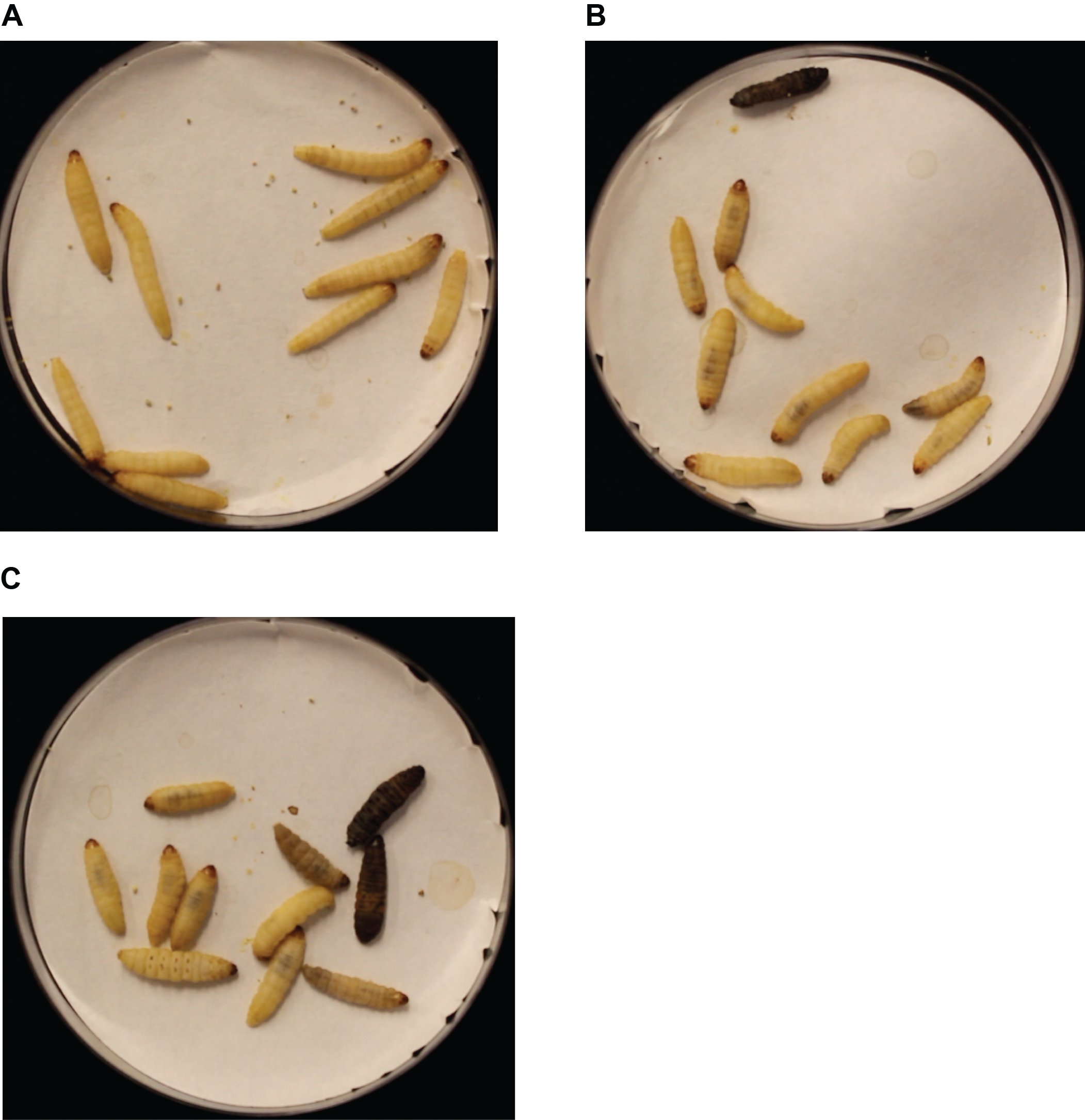

Supplement: S3 Fig — (A) Photograph of last instar waxworm larvae 5 days after injection with PBS. (B) Photograph of last instar waxworm larvae 5 days after injection with 4 μg of venom collected from axenic S. carpocapsae IJs that had been activated for 12 h in waxworm homogenate. (C) Another photograph of last instar waxworm larvae 5 days after injection with 4 μg of venom collected from axenic S. carpocapsae IJs that had been activated for 12 h in waxworm homogenate. (TIF) [file ppat.1006302.s003.tif]

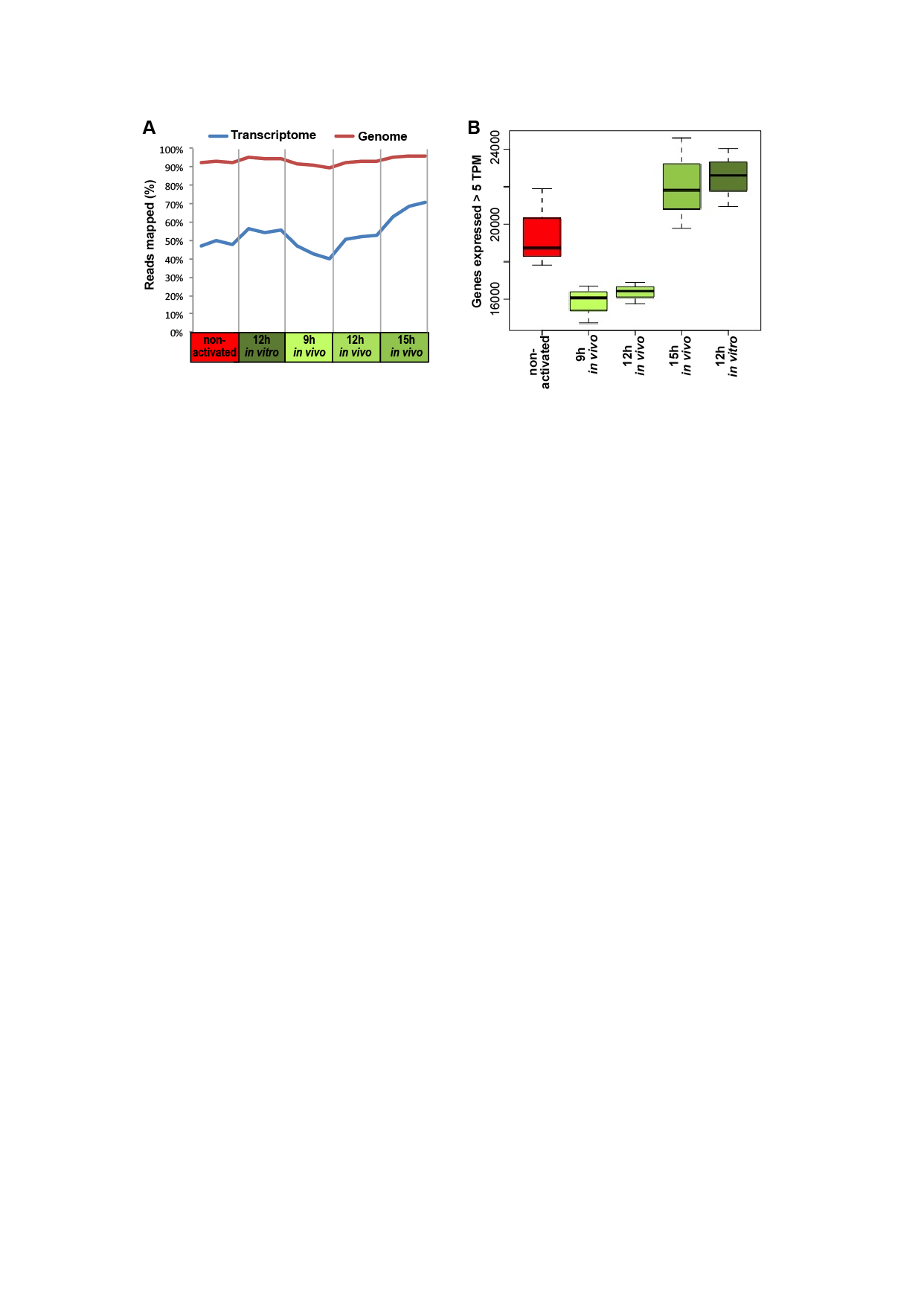

Supplement: S4 Fig — (A) Plot showing the percentage of reads mapped to the S. carpocapsae transcriptome and genome for each individual IJ RNA-seq sample. Samples are grouped based on their activation stages or methods of activation. (B) Boxplot showing the numbers of genes expressed greater than 5 counts for each activation stage and method of activation. (TIF) [file ppat.1006302.s004.tif]

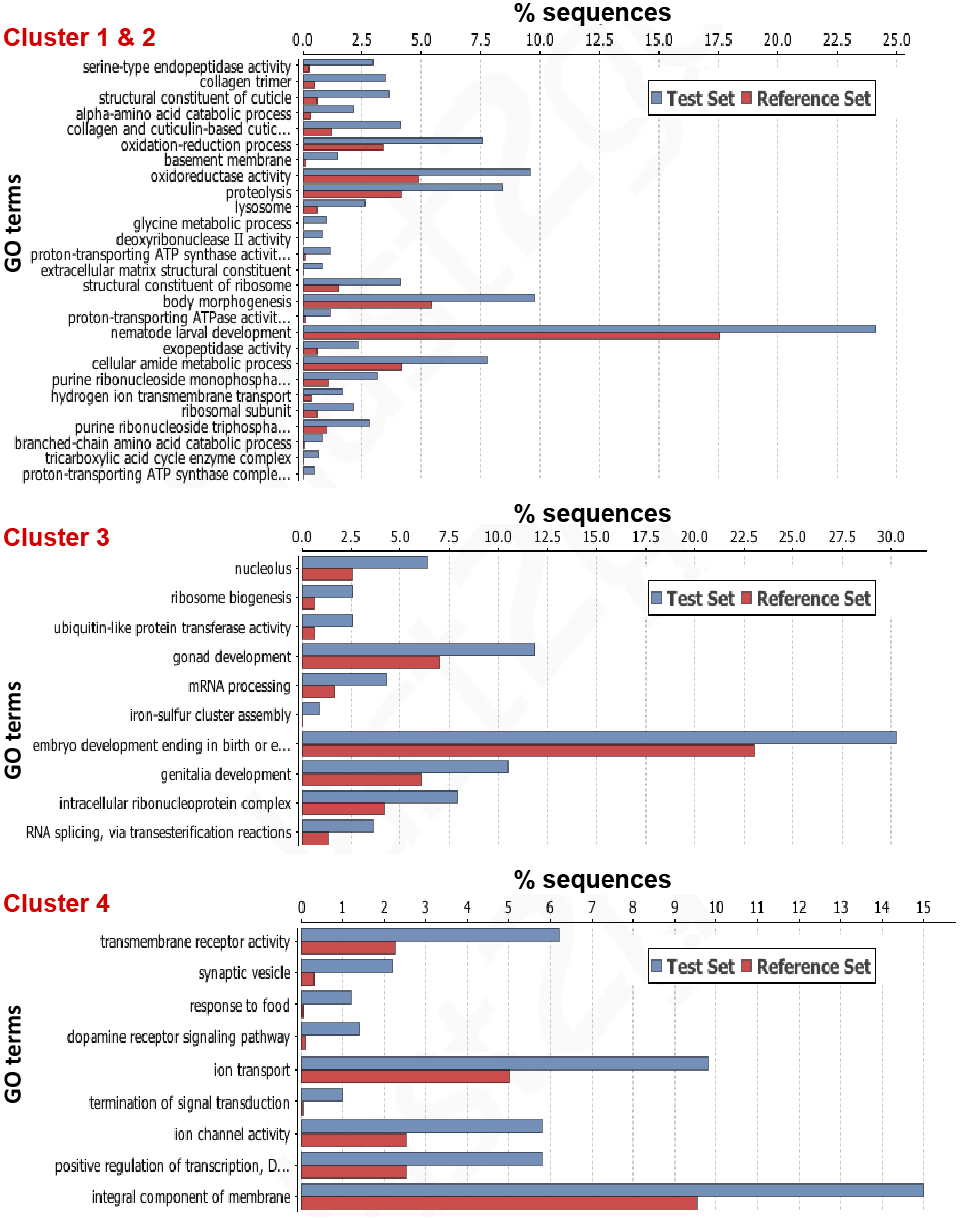

Supplement: S5 Fig — (TIF) [file ppat.1006302.s005.tif]

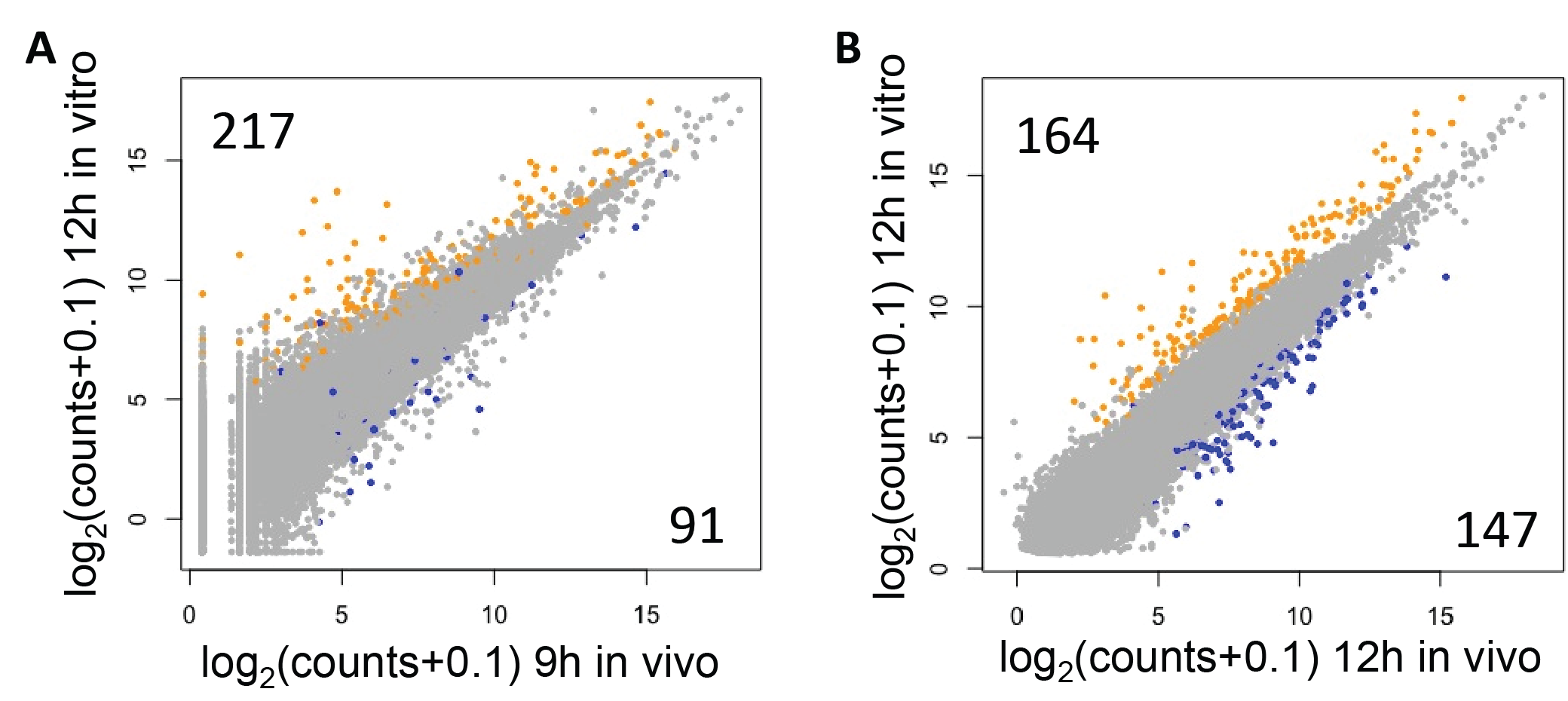

Supplement: S6 Fig — (A) Comparison of the transcriptome of 12h in vitro activated IJs with that of 9h in vivo activated IJs. (B) Comparison of the transcriptome of 12h in vitro activated IJs with that of 12h in vivo activated IJs. (TIF) [file ppat.1006302.s006.tif]

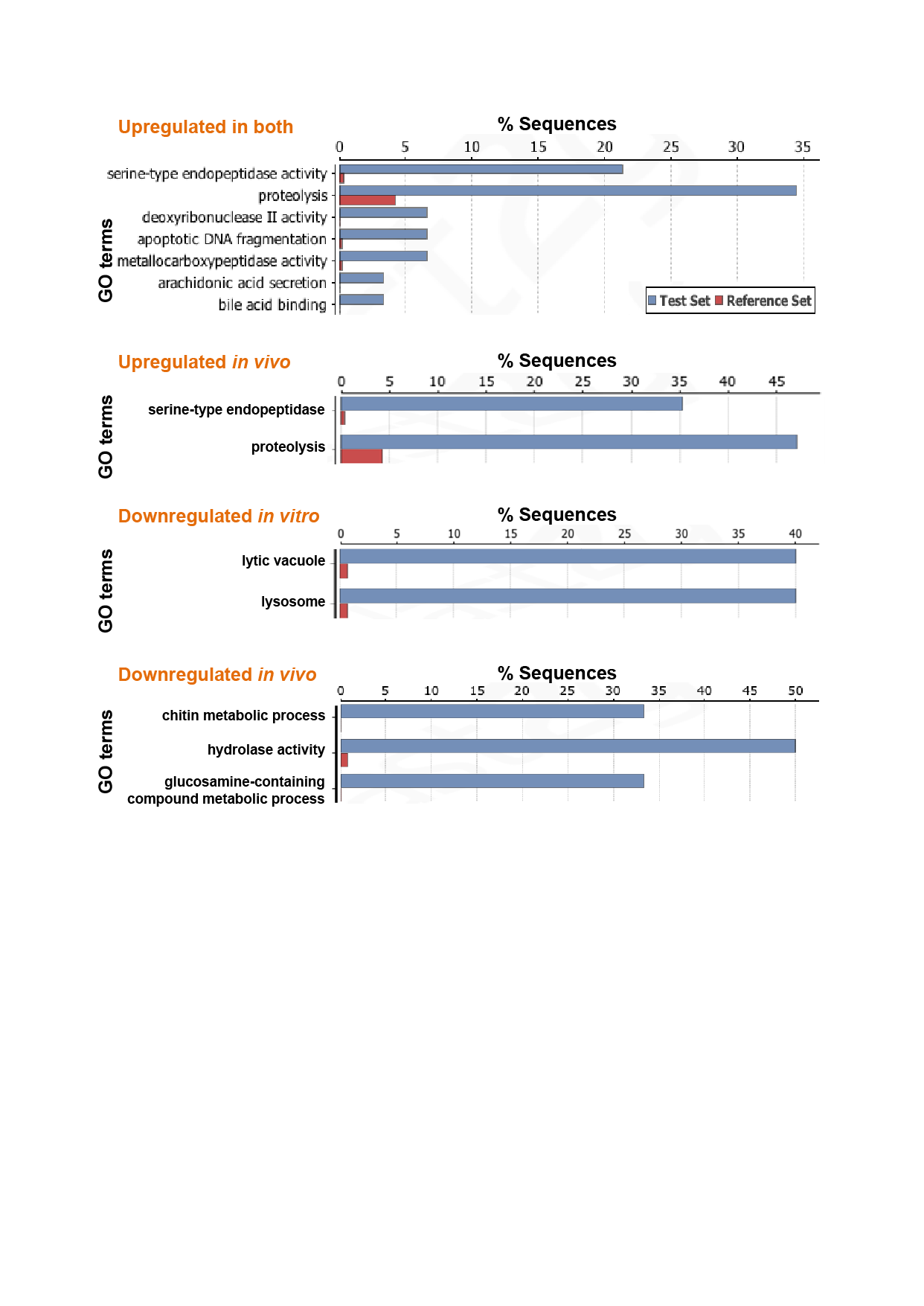

Supplement: S7 Fig — (TIF) [file ppat.1006302.s007.tif]

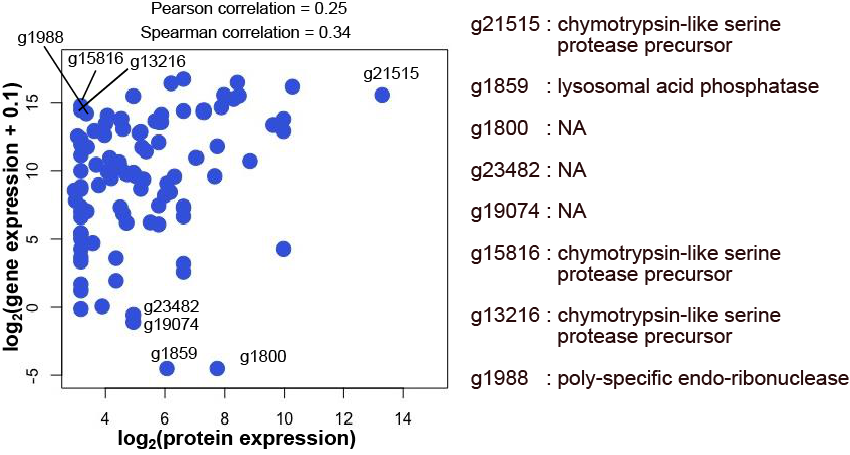

Supplement: S8 Fig — A few examples of proteins with different types of RNA-Protein correlations are shown on the right. g21515; g23482 and g19074 have good RNA-Protein correlation; g1859 and g1800 have high protein and low RNA levels; g1988; g15816 and g13216 are examples for those with high RNA and low protein levels. (TIF) [file ppat.1006302.s008.tif]

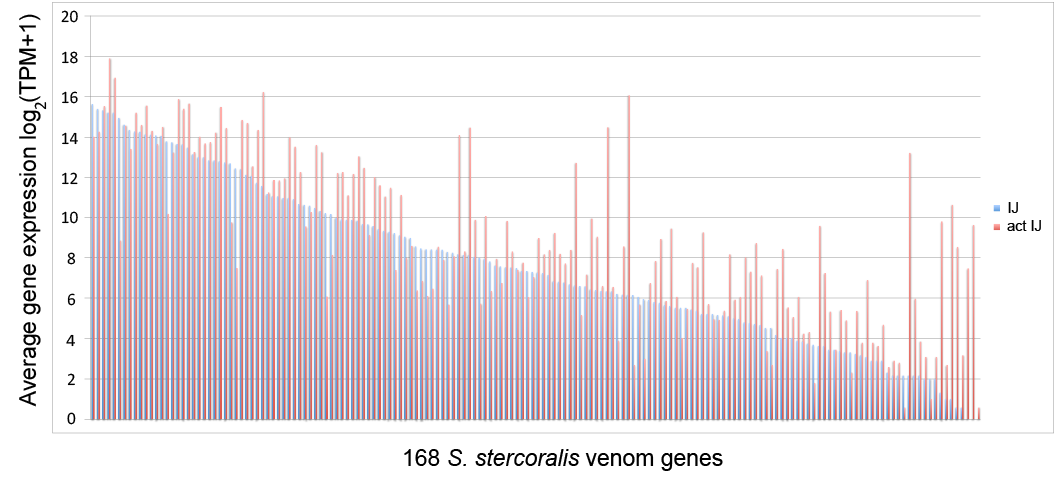

Supplement: S9 Fig — A bar graph showing the expression levels of 168 venom protein orthologs in S. stercoralis. Blue bars shows the expression of these genes in the infectious 3rd larval stage, equivalent to S. carpocapsae IJs. Red bars show the expression of these genes in tissue migrating 3rd larval stage, equivalent to activated or activating S. carpocapsae IJs. RNA-seq data came from previously published work [54]. (TIF) [file ppat.1006302.s009.tif]
