# Supplementary material for: Activated entomopathogenic nematode infective juveniles release lethal venom proteins
Source: PLoS Pathog. 2017 Apr 20;13(4):e1006302. doi: 10.1371/journal.ppat.1006302 (PMC5398726; doi:10.1371/journal.ppat.1006302)
Supplement: S1 Table — (DOCX) [file ppat.1006302.s010.docx]

S1 Table. The percentage of exsheathed IJs without activation.

| **replicates** | **exsheathed IJs** | **ensheathed IJs** | **% exsheathed** |
| --- | --- | --- | --- |
| **IJs_1** | 47 | 77 | 37.90 |
| **IJs_2** | 40 | 97 | 29.20 |
| **IJs_3** | 36 | 91 | 28.35 |
| **IJs_4** | 44 | 103 | 29.93 |
| **Average** | **41.75** | **92** | **31.34** |
