# Supplementary material for: Activated entomopathogenic nematode infective juveniles release lethal venom proteins
Source: PLoS Pathog. 2017 Apr 20;13(4):e1006302. doi: 10.1371/journal.ppat.1006302 (PMC5398726; doi:10.1371/journal.ppat.1006302)
Supplement: S2 Table — (DOCX) [file ppat.1006302.s011.docx]

S2 Table. *S. carpocapsae* IJ time course activation rates.

| **Replicates** | **Fully activated#** | **Partially activated#** | **Non-activated#** | **Sum#** | **Fully activated (%)** | **Partially activated (%)** | **Non-activated (%)** |
| --- | --- | --- | --- | --- | --- | --- | --- |
| **6hr_1** | 147 | 814 | 184 | 1145 | 12.8384279 | 71.09170306 | 16.069869 |
| **6hr_2** | 103 | 535 | 114 | 752 | 13.6968085 | 71.14361702 | 15.1595745 |
| **6hr_3** | 107 | 571 | 67 | 745 | 14.3624161 | 76.6442953 | 8.99328859 |
| **Average** | 119 | 640 | 121.6667 | 880.6667 | 13.5124905 | 72.67221802 | 13.8152914 |
| Standard error | | | | | 0.36016409 | 1.504209368 | 1.81485341 |
| **Replicates** | **Fully activated#** | **Partially activated#** | **Non-activated#** | **Sum#** | **Fully activated (%)** | **Partially activated (%)** | **Non-activated (%)** |
| **12hr_1** | 283 | 302 | 21 | 606 | 46.69967 | 49.8349835 | 3.46534653 |
| **12hr_2** | 301 | 485 | 42 | 828 | 36.352657 | 58.57487923 | 5.07246377 |
| **12hr_3** | 465 | 602 | 68 | 1135 | 40.969163 | 53.03964758 | 5.99118943 |
| **Average** | 349.6667 | 463 | 43.66667 | 856.3333 | 40.833009 | 54.06773063 | 5.09926041 |
| Standard error | | | | | 2.4435214 | 2.084283682 | 0.60267198 |
| **Replicates** | **Fully activated#** | **Partially activated#** | **Non-activated#** | **Sum#** | **Fully activated (%)** | **Partially activated (%)** | **Non-activated (%)** |
| **18hr_1** | 488 | 522 | 91 | 1101 | 44.3233424 | 47.41144414 | 8.26521344 |
| **18hr_2** | 493 | 575 | 26 | 1094 | 45.0639854 | 52.55941499 | 2.37659963 |
| **18hr_3** | 543 | 531 | 25 | 1099 | 49.4085532 | 48.3166515 | 2.27479527 |
| **Average** | 508 | 542.666667 | 47.33333 | 1098 | 46.2659381 | 49.42319369 | 4.31086825 |
| Standard error | | | | | 1.29505028 | 1.295605768 | 1.61670958 |
| **Replicates** | **Fully activated#** | **Partially activated#** | **Non-activated#** | **Sum#** | **Fully activated (%)** | **Partially activated (%)** | **Non-activated (%)** |
| **24hr_1** | 508 | 364 | 11 | 883 | 57.5311438 | 41.22310306 | 1.24575311 |
| **24hr_2** | 394 | 364 | 15 | 773 | 50.9702458 | 47.08926261 | 1.94049159 |
| **24hr_3** | 507 | 402 | 12 | 921 | 55.0488599 | 43.64820847 | 1.3029316 |
| **Average** | 469.6667 | 376.666667 | 12.66667 | 859 | 54.6759798 | 43.84943733 | 1.47458285 |
| Standard error | | | | | 1.56160186 | 1.389561852 | 0.18180308 |
| **Replicates** | **Fully activated#** | **Partially activated#** | **Non-activated#** | **Sum#** | **Fully activated (%)** | **Partially activated (%)** | **Non-activated (%)** |
| **30hr_1** | 263 | 202 | 5 | 470 | 55.9574468 | 42.9787234 | 1.06382979 |
| **30hr_2** | 334 | 245 | 7 | 586 | 56.996587 | 41.80887372 | 1.19453925 |
| **30hr_3** | 451 | 374 | 9 | 834 | 54.0767386 | 44.8441247 | 1.07913669 |
| **Average** | 349.3333 | 273.666667 | 7 | 630 | 55.4497354 | 43.43915344 | 1.11111111 |
| Standard error | | | | | 0.69767844 | 0.721649828 | 0.03368537 |

p-values (ttest) <0.01 are considered as significant differences

|  | **Fully activated rate** | **Partially activated rate** | **Non-activated rate** |
| --- | --- | --- | --- |
| **6hr-12hr** | 0.000788686 | 0.00369615 | 0.02164091 |
| **6hr-18hr** | 3.82157E-05 | 0.00063791 | 0.03774354 |
| **6hr-24hr** | 3.13882E-05 | 0.00032072 | 0.00595678 |
| **6hr-30hr** | 1.63613E-06 | 0.00012943 | 0.00522259 |
| **12hr-18hr** | 0.219616868 | 0.2181547 | 0.81177195 |
| **12hr-24hr** | 0.020655583 | 0.03277824 | 0.01224562 |
| **12hr-30hr** | 0.009982684 | 0.01715932 | 0.0072505 |
| **18hr-24hr** | 0.029352931 | 0.07947204 | 0.231395 |
| **18hr-30hr** | 0.0064101 | 0.02668908 | 0.18220215 |
| **24hr-30hr** | 0.609186217 | 0.7063345 | 0.16527496 |
