# Supplementary material for: Activated entomopathogenic nematode infective juveniles release lethal venom proteins
Source: PLoS Pathog. 2017 Apr 20;13(4):e1006302. doi: 10.1371/journal.ppat.1006302 (PMC5398726; doi:10.1371/journal.ppat.1006302)
Supplement: S3 Table — (DOCX) [file ppat.1006302.s012.docx]

| **replicates** | **number of IJs** | **total venom secreted in 3hrs (µg)** | **venom secreted in 3hrs per IJ (ng/IJ)** | **extrapolation to 24hrs venom secretion (ng/IJ)** | **number of IJs required for secreting 10ng venom in 24hrs** |
| --- | --- | --- | --- | --- | --- |
| **(1) Sc 12hr** | 2 million | 105.376 | 0.053 | 0.421503417 | 23.7 |
| **(2) Sc 12hr** | 2 million | 94.907 | 0.047 | 0.379626424 | 26.3 |
| **(3) Sc 12hr** | 0.2 million | 14.241 | 0.071 | 0.569658314 | 17.5 |
| **(4) Sc 12hr** | 2 million | 144.171 | 0.072 | 0.576683371 | 17.3 |
| **(5) Sc 12hr** | 2 million | 120.928 | 0.060 | 0.483712073 | 20.6 |
| **(6) Sc 12hr** | 2 million | 120.977 | 0.060 | 0.483908884 | 20.6 |
| **Average** |  | **100.100** | **0.061** | **0.485848747** | **20.5** |

S3 Table. Estimated venom amount per IJ and the number of IJs required for secreting enough venom (10 ng) to kill *Drosophila*.
