# Supplementary material for: Activated entomopathogenic nematode infective juveniles release lethal venom proteins
Source: PLoS Pathog. 2017 Apr 20;13(4):e1006302. doi: 10.1371/journal.ppat.1006302 (PMC5398726; doi:10.1371/journal.ppat.1006302)
Supplement: S5 Table — (DOCX) [file ppat.1006302.s014.docx]

**S5 Table.** Orthology Clusters

| Species name | Stst | Tc | Bm | As | Ac | Od | Hc | Dv | Hb | Ss | Sf | Sg | Sm |
| --- | --- | --- | --- | --- | --- | --- | --- | --- | --- | --- | --- | --- | --- |
| # of clusters | 116 | 108 | 80 | 98 | 130 | 121 | 94 | 69 | 93 | 296 | 225 | 217 | 216 |

*Strongyloides stercoralis* (Stst), *Toxocara canis* (Tc), *Brugia malayi* (Bm), *Ascaris suum* (As), *Ancylostoma ceylanicum* (Ac), *Oesophagostomum detatum* (Od), *Haemonchus contortus* (Hc), *Dictyocaulus vivparus* (Dv), *Heterhabditis bacteriophora* (Hb), *Steinernema scapterisci* (Ss), *S. feltiae* (Sf), *S. glaseri* (Sg), *S. monticolum* (Sm).
